# Soil microbial effects on tree seedling performance during forest secondary succession

**DOI:** 10.1002/ecy.70477

**Published:** 2026-09-04

**Authors:** Anita Weissflog, Bettina M. J. Engelbrecht, John R. Healey, Lars Markesteijn

**Affiliations:** ^1^ School of Environmental and Natural Sciences, Bangor University Bangor UK; ^2^ School of the Environment, Yale University New Haven Connecticut USA; ^3^ Yale Institute for Biospheric Studies Yale University New Haven Connecticut USA; ^4^ Department of Plant Ecology, Bayreuth Center of Ecology and Environmental Research (BayCEER) University of Bayreuth Bayreuth Germany; ^5^ Smithsonian Tropical Research Institute Apartado Panama; ^6^ Global Change Research Institute (IICG‐URJC) Universidad Rey Juan Carlos Móstoles Spain; ^7^ Department of Biology Universidad Rey Juan Carlos (URJC) Móstoles Spain; ^8^ Present address: Department of Environmental Systems Science ETH Zurich Zurich Switzerland

**Keywords:** forest recovery, microbiota, plant–microbial interactions, plant–soil feedback, rainforest, secondary succession, seedling performance, soil fungi

## Abstract

Soil microbiota influence seedling survival and abundance in mature forests. However, their role in tree species turnover during secondary forest succession remains unclear, especially in species‐rich tropical forests. We conducted a shade house experiment to assess variation in the direction and strength of net soil microbial effects on seedling emergence, biomass growth, and mortality of seven tree species. We tested for effects of soil successional stage (0, 15, 25, and 115 years of forest recovery), tree species' association with successional stages (home stages where species peak in abundance vs. away stages), and light level (5% vs. 40% of daylight). We quantified net microbial effects as the ratio of seedling performance in live soil (including microbiota) versus sterilized and fungicide‐treated soil. We found net positive or net negative microbial effects on seedling performance in six of the seven tree species, highlighting the prevalence of microbial effects. Overall, fewer seedlings emerged and more died in live than sterilized soils, and under high than low‐light levels, although these effects varied greatly among tree species. We found no effect of soil successional stage on seedling performance. Tree species' association with successional stages, however, strongly affected seedling performance: Soil microbiota from a species' home successional stage increased the likelihood of seedling emergence by approximately 16% and reduced seedling mortality by approximately 36%. Our results suggest that soil microbiota exert tree species‐specific effects on seedling performance during secondary succession of tropical forest. Positive microbial effects on seedling emergence and survival in tree species' home successional stages may promote tree species' establishment and persistence at home stages and could thereby slow tree species turnover during forest recovery. While the generality of our results needs to be tested across forest ecosystems, they highlight the relatively unexplored role of soil microbiota in determining the pace and direction of successional plant community assembly and promote the development of microbial inoculation as a tool for restoration of functionality and biodiversity of forests globally.

## INTRODUCTION

Secondary successional forests are an increasingly common feature of many landscapes (FAO, 2020) and offer the most cost‐effective nature‐based solution to climate change through carbon sequestration while simultaneously restoring biodiversity (Chazdon et al., 2016; Lewis et al., 2019). However, we still do not fully understand the mechanisms that drive the recovery of tree species composition (Rozendaal et al., 2019). While research on forest succession has traditionally emphasized competition and aboveground drivers such as light availability and precipitation, belowground biotic interactions have received comparatively less attention. Soil microbiota affect tree species' local abundance and community composition in mature forests (Bagchi et al., 2014; Segnitz et al., 2020; Tedersoo et al., 2020) and play a critical role in forest recovery by improving nitrogen fixation (Batterman et al., 2013) and plant access to nutrients (Baldrian et al., 2023), enabling plant growth on nutrient‐poor, degraded soils. Microbial inoculation has therefore been proposed as a promising tool to promote forest recovery (Averill et al., 2022). However, recommendations applicable to forests do not exist because of our limited knowledge of the specificity of plant–microbial interactions and their modulation by abiotic conditions, especially in species‐rich forests (Averill et al., 2022; Baldrian et al., 2023). An understanding of how soil microbiota interact with tree species across successional stages is thus needed to advance knowledge of plant community assembly and has important implications for the development of effective forest restoration approaches.

Seeds and seedlings initiate forest recovery and are vulnerable life stages that may be particularly sensitive to positive and negative microbial effects (Mangan et al., 2010; Sarmiento et al., 2017). In tropical moist forests, first‐year seedling mortality through fungal pathogens is extremely high (Augspurger, 1984) and approximately 5000 seeds are needed to establish a single sapling (Condit et al., 2025). Enormous variation in tree species' mortality (Condit et al., 2025) under different environmental conditions is thought to be a main driver of the shift in dominance from light‐demanding to shade‐tolerant tree species during forest succession (Craven et al., 2015; Finegan, 1996). Understanding tree species‐specific soil microbial effects on seedling performance across succession is essential to assess the microbiome's role in tree community assembly and guide microbial inoculation as a potentially powerful tool for forest restoration.

Secondary succession of tropical forests is characterized by a turnover of tree species (Finegan, 1996; Rüger et al., 2023; van Breugel et al., 2013) that are associated with particular stages of succession at which they peak in abundance, herein referred to as species' “home” stages. Soil microbial community composition is strongly influenced by the identity of the dominant tree species in mature temperate (Urbanová et al., 2015) and tropical forests (Schroeder et al., 2019). Soil fungal pathogens reflect tree community composition more strongly than mutualist fungi (Schroeder et al., 2019), and a higher host seedling density facilitates pathogen build‐up and transmission (Liu et al., 2015). An accumulation of more host‐specific pathogenic fungi may thus amplify negative microbial effects for tree species at their home successional stages and thereby drive successional tree species turnover.

Tree life history strategy may affect microbial effects on seedlings. Fast‐growing species that dominate in early stages of forest succession (“early‐successional species”) (Rüger et al., 2023) typically invest less in defense (Coley & Barone, 1996) and, as a consequence, may be more susceptible to pathogens than slow‐growing, better‐defended species characteristic of later‐successional stages (“late‐successional species”). Indeed, in agricultural soils, microbial effects on the growth of early‐successional trees were negative, while microbial effects on slower growing tree species were neutral (Pizano et al., 2014). Negative microbial effects on early‐successional species and positive effects on late‐successional species are thought to drive species turnover in grassland succession (Kardol et al., 2006; van der Putten et al., 2013). Whether similar patterns apply to forests remains, however, unclear.

The direction and strength of microbial effects may change with forest successional stage due to changes in abiotic conditions and tree species richness. The decrease in light level below the canopy (Matsuo et al., 2021) may affect plant–microbe interactions in particular (Xi et al., 2023). High light levels benefit microbial mutualists by enhancing sporulation (Mangan et al., 2010) and promoting mycorrhizal colonization through altered plant physiology (Kiers et al., 2011). Indeed, mycorrhizal abundance was found to be highest in high light, early‐successional stages and decreased during forest recovery (Zangaro et al., 2012). Fungal pathogens, in contrast, infect a higher proportion of seeds (Pringle et al., 2007) and cause higher seedling mortality under low‐light levels (Augspurger, 1984), potentially leading to an increase in the strength of pathogen effects as understory light decreases during succession. This process could be compounded by the successional increase in tree species richness (Poorter et al., 2021) and the high host specificity of pathogens (Schroeder et al., 2019) and may explain the higher diversity and abundance of fungal pathogens in forest soils of later‐successional stages (Saltonstall et al., 2025). Indeed, fungal pathogen impacts on seedling communities were found to be negligible in the very early stages of forest succession (Szefer et al., 2020) but affect seedling dynamics in mature forests (e.g., Bagchi et al., 2014). Net microbial effects may therefore be positive early in succession and become increasingly negative with forest successional stage.

Soil microbiota may play a pivotal, yet largely overlooked, role in the secondary succession of forests. In a shade house experiment, we assessed variation in net soil microbial effects on seedling performance with tree species identity, forest successional stage, tree species association with successional stages, and light level over the first 115 years of forest recovery in Panama. We hypothesized that (1) tree species experience the strongest negative microbial effects in soils from their “home” stage, that is, the successional stage(s) where they are most abundant, (2) microbial effects are more negative for early‐successional than for late‐successional tree species, (3) microbial effects increase in strength over the course of forest recovery, and that (4) microbial effects are more positive under higher light levels.

## METHODS

### Study site and experimental design

**FIGURE 1:**
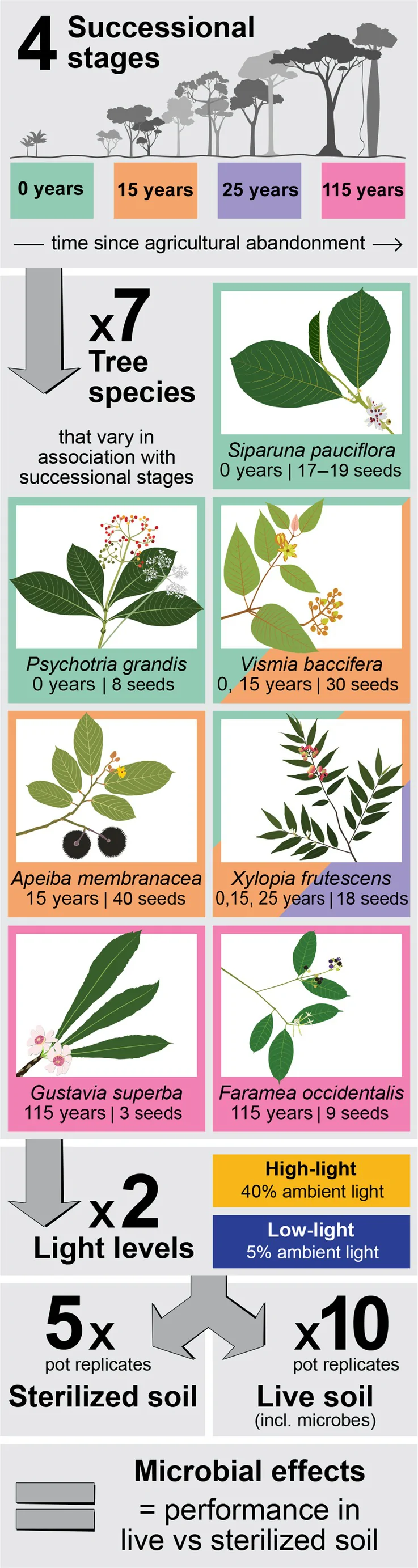
Full‐factorial experimental design. Seeds of seven tree species were sown in soils from secondary forests of four successional stages. Tree species are associated with different home stages, at which they are among the 20 most abundant tree species. Home stages and the number of seeds per pot are printed below each species name. Seedlings were grown under two light levels. Microbial effects are the difference in performance of seedlings grown in live forest soil (including microbiota) versus sterilized and fungicide‐treated forest soil. All illustrations created by Anita Weissflog.

This study was performed at the Smithsonian Tropical Research Institute in Gamboa (9° 07′ N, 79° 42′ W), Panama, using seeds and soils from semideciduous moist lowland forests within and near Soberanía National Park. These forests receive 2300–2700 mm rain per year, mostly during the wet season from May to December (Paton, 2020). In a fully factorial shade house experiment (Figure 1), we quantified the effects of soil microbiota (live vs. sterilized and fungicide‐treated soils) collected from forests of four successional stages (0, 15, 25, and 115 years of forest recovery) under two light levels (40% vs. 5% of daylight) on seedling emergence, biomass (as a measure of growth), and mortality in seven tree species. Tree species varied in their abundance at, and thus their association with, the four successional stages (home vs. away). Seeds were sown between November 2018 and June 2019 and seedlings were harvested in August 2020 (Appendix S1: Table S1).

### Tree species selection and seed collection

To include tree species that are typical of different successional stages of Panamanian moist forests, we selected species based on their relative abundance in our successional stages (Figure 1). We calculated the importance value (IV) based on census data from forests across Panama (Condit et al., 2019; Condit, 1998; M. van Breugel unpublished data) as the sum of relative basal area (basal area of trees >1 cm dbh of a species divided by the basal area of all trees per site) and relative density (number of trees of a species divided by the number of all trees per site) divided by two (Schroeder et al., 2019). Based on the IV, we preselected the 20 most abundant species per stage. Among those, we selected seven species for which seeds were available for field collection. All our species form associations with mutualistic arbuscular mycorrhizas (Appendix S1: Section S1). To avoid preexperimental infection with soil microbiota, we collected seeds directly from trees or intact fruits on the forest floor. For each species, we collected seeds from six or more trees located > 4 km away from all soil extraction sites to limit bias from plant genotype‐specific pathogens or adaptation to local microbiomes (Eck et al., 2019). We cleaned, dried, and pooled undamaged seeds per species. Seeds were sown within 30 days of collection, except *Apeiba membranacea* Spruce ex Benth. seeds that were stored for ≤103 days. Germination of *A. membranacea* has been shown to be high, at approximately 90%, after 2 years of storage (Zalamea et al., 2015). All seeds were soaked in water for 24 h and surface‐sterilized (70% ethanol, 10% bleach, distilled water) directly before sowing. Seed number per pot varied among species depending on seed size (Figure 1).

### Soil collection and inoculum preparation

To experimentally isolate the effects of soil microbiota while minimizing variation in soil physicochemical properties, soils from different successional stages were used as inoculum added to a standardized, steam‐sterilized background soil (see below). Because several soil properties and plant community characteristics recover within the first decades of forest succession (Poorter et al., 2021; Rozendaal et al., 2019), we included three young (0, 15, 25 years) and one old (115 years) successional stage. Soils of the young stages were collected in the Agua Salud landscape, a matrix of agricultural fields and secondary forests of different successional stages (9°13′ N, 79°47′ W; van Breugel et al., 2013) with a similar land‐use history (cattle pasture and low‐intensity agriculture; Craven et al., 2015). Soils are Oxisols with a mean pH of approximately 5.0 that vary little in texture and nutrients (van Breugel et al., 2013). Soils of the old successional stage were collected in a nearby forest (9°9′ N 79°44′ W; Condit, 1998; Appendix S1: Figure S1) growing on a mosaic of Oxisols and Alfisols (Turner & Engelbrecht, 2011) with no recorded anthropogenic disturbance since logging and farming in the early 20th century. We collected a total of 10 soil samples obtained from two locations (50 m apart) in each of five forest sites (≥ 1 km apart) per successional stage. The sites were separated by forests of different successional stages, except the 115‐year‐old soils that were collected in a continuous forest. At each sampling location, we removed surface litter and extracted a 25 × 50 cm sample of the top 0–10 cm of soil. All 10 soil samples of a successional stage were pooled, sieved (4 mm), and thoroughly mixed, as our objective was to compare effects of the microbiota that are typical of each successional stage rather than within‐stage variation (Appendix S1: Section S1). Pooled soils were stored in a ventilated, dark room until being used within 22 days of collection to inoculate experimental pots either “live” (incl. microbiota) or “sterilized” (autoclaved 2 × 57 min at 121°C).

Using a small amount of inoculum minimizes potential bias from successional changes in soil pH and nutrients, and nutrient pulses through microbial lysis after sterilization, while effectively introducing soil microbiota (Mangan et al., 2010). We thus filled all pots (ø 21 cm, 4.1‐L volume) to 90% with the same “background soil”: a steam‐sterilized (2 × 2.5 h at ≥75°C) 1:1 mix of sand and soil (homogenous mix of Oxisols from various 60–90‐year‐old forests in Panama). We then added 400 mL of either live or sterilized successional forest soil inoculum as a surface layer. Differences between live and sterilized pots thus reflect differences in the inoculum, even if some microbiota survive steam sterilization of the background soil and soil properties may be altered (Dietrich et al., 2020). Space and resource limitations necessitated uneven replicate numbers between the live and sterilized soils. Because plant–microbe interactions were expected to increase within‐treatment variation in plant performance, we used 10 live and 5 sterilized replicates per species‐treatment combination.

### Shade house set up

The experiment comprising 840 pots (7 species × 4 successional stages × 2 light levels × soil status (10 live + 5 sterilized replicates); Figure 1) was set up in a shade house, which was entirely covered in standardized shade cloth eliminating 60% of daylight. Light level at bench height was thus at 40% of daylight and constituted the high light level. To create the 5% low‐light conditions similar to those in forest understory, we systematically covered individual tables with additional shade cloth. Each table was divided into a section for pots with live soil and a section for pots with sterilized soil by a 1‐m tall clear plastic sheet to prevent fungicide transfer or cross‐infection. Pot position was randomly assigned with no more than two pots per species‐treatment combination per table (Appendix S1: Figure S2).

Sterilized soils are rapidly recolonized by microbiota, especially in open shade house experiments (Bollen, 1974; Mukjang et al., 2022). To continuously suppress fungal growth in sterilized soils, we thus subsequently applied the systemic fungicide Amistar XTRA (200 g L^−1^ Azoxystrobin, 80 g L^−1^ Cyproconazole; Syngenta) to soil and seedlings according to the manufacturer's guidelines (575 mL ha^−1^, every 6 weeks). Amistar XTRA is active against Oomycetes, Ascomycetes, Basidiomycetes, and Deuteromycetes and thus against all the most common pathogens of seedlings in these forests (Spear & Broders, 2021).

Species were sown at different times due to different seed production times (Appendix S1: Table S1). Seeds were placed onto the inoculum layer (between 3 and 40 seeds per pot; Figure 1). We watered every other day, avoided wetting of leaves, and removed shed leaves. To limit competition, we thinned seedlings to a maximum of three seedlings per pot 7 months after sowing. We recorded seedling emergence and survival every other day. To assess growth, we measured dry biomass by harvesting, oven‐drying (>72 h at 60°C), and weighing (*d*: 0.0001 g) all 793 seedlings alive at the end of the experiment (including roots, stems, and leaves, excluding abscised leaves).

### Statistical analyses

We analyzed the effects of inoculum (live vs. sterilized), species (seven species), soil successional stage (0, 15, 25, 115 years of recovery) and light (40% vs. 5% daylight) on seedling emergence, biomass growth, and mortality. For each response variable, we performed two separate analyses using either (a) successional stage or (b) tree species' association with successional stages. The binary variable association statistically pools successional stages where a species is among the 20 most abundant species into “home” and pools all other stages into “away.” Because some tree species were abundant at multiple successional stages, the association analysis allows us to evaluate the importance of home microbiota independent of recovery time. Hereafter, “successional stage/association” refers to conducting separate analyses. We determined the random effects structure of each model using Akaike's information criterion (AIC) and included an effect if it improved model fit by delta‐AIC >4 (Burnham & Anderson, 2004).

### Emergence

To analyze variation in emergence, we used generalized linear mixed‐effects models with binomial errors and a bobyqa optimizer (Bates et al., 2015). We modeled the probability of seedling emergence as a function of inoculum, species, successional stage/association, and light. To assess variation in microbial effects with species, successional stage/association, and light, models included their two‐way interaction terms with inoculum. To account for spatial heterogeneity in the shade house, models included seedling position (table and pot identifiers) as random effects. We calculated microbial effects as logarithmic odds ratios (log(OR)) of emergence in live versus sterilized soils for each species, successional stage, association, and light level (Lenth, 2022). A log(OR) > 0 indicates a positive microbial effect, that is, higher emergence in live than sterilized soil. We compared microbial effects between species, successional stage/association, and light levels with Bonferroni‐adjusted pairwise contrasts (Lenth, 2022).

### Biomass growth

We analyzed variation in biomass growth using linear mixed‐effects models (Bates et al., 2015). We modeled biomass growth as a function of inoculum, species, and successional stage/association and the interactions between inoculum and species, and between inoculum and successional stage/association. Except for *A. membranacea*, *Gustavia superba* (Kunth) O. Berg, and *Faramea occidentalis* (L.) A. Rich., all seedlings growing under 5% light died before harvest precluding the analysis of the effect of light as a fixed effect. We thus included light and table identifier as predictors. To account for different seedling ages and densities, we included the standardized number of days between emergence and harvest and the standardized number of seedlings per pot at harvest. We calculated microbial effects as the predicted difference in mean biomass growth between live and sterilized soil.

### Mortality

We analyzed seedling mortality over 415 days after sowing (maximum experimental time for the last sown species) with Cox proportional hazard regressions (Therneau, 2024). We modeled mortality as a function of inoculum, species, successional stage/association, and light level, and the two‐way interactions between inoculum and each of the other main effects. To account for different seedling densities, we included the standardized number of emerged seedlings per pot together with table and pot identifiers as random effects. Because the effects of inoculum and light varied over time and thereby violated the proportional hazard assumption, we split our data into five 83‐day intervals (Gordon & Seifert, 2022) and added the interaction terms of inoculum and light with time. We assessed the hazard ratio (HR), that is, the probability of mortality in the next time interval, in live versus sterilized soils per species, successional stage/association, and light level. A HR >1 translates into a higher mortality risk in live than sterilized soil, that is, a negative microbial effect on survival. We compared microbial effects among species, successional stage/association, and light levels with Bonferroni‐corrected pairwise contrasts. We excluded *G. superba* from the analysis because no *G. superba* seedling died in the sterilized soils and Cox models cannot handle groups with zero deaths. We show Kaplan–Meier curves (Kassambara et al., 2021) for all species.

For all models, we tested for the normality of residuals, homoscedasticity, and non‐multicollinearity (Lüdecke et al., 2021) and performed type III ANOVAs (Fox & Weisberg, 2019). All analyses were conducted in R 4.2.2 (R Core Team, 2024). Information on R packages and additional analyses at the species level is provided in Appendix S1 and S2, respectively.

## RESULTS

### Emergence

**FIGURE 2:**
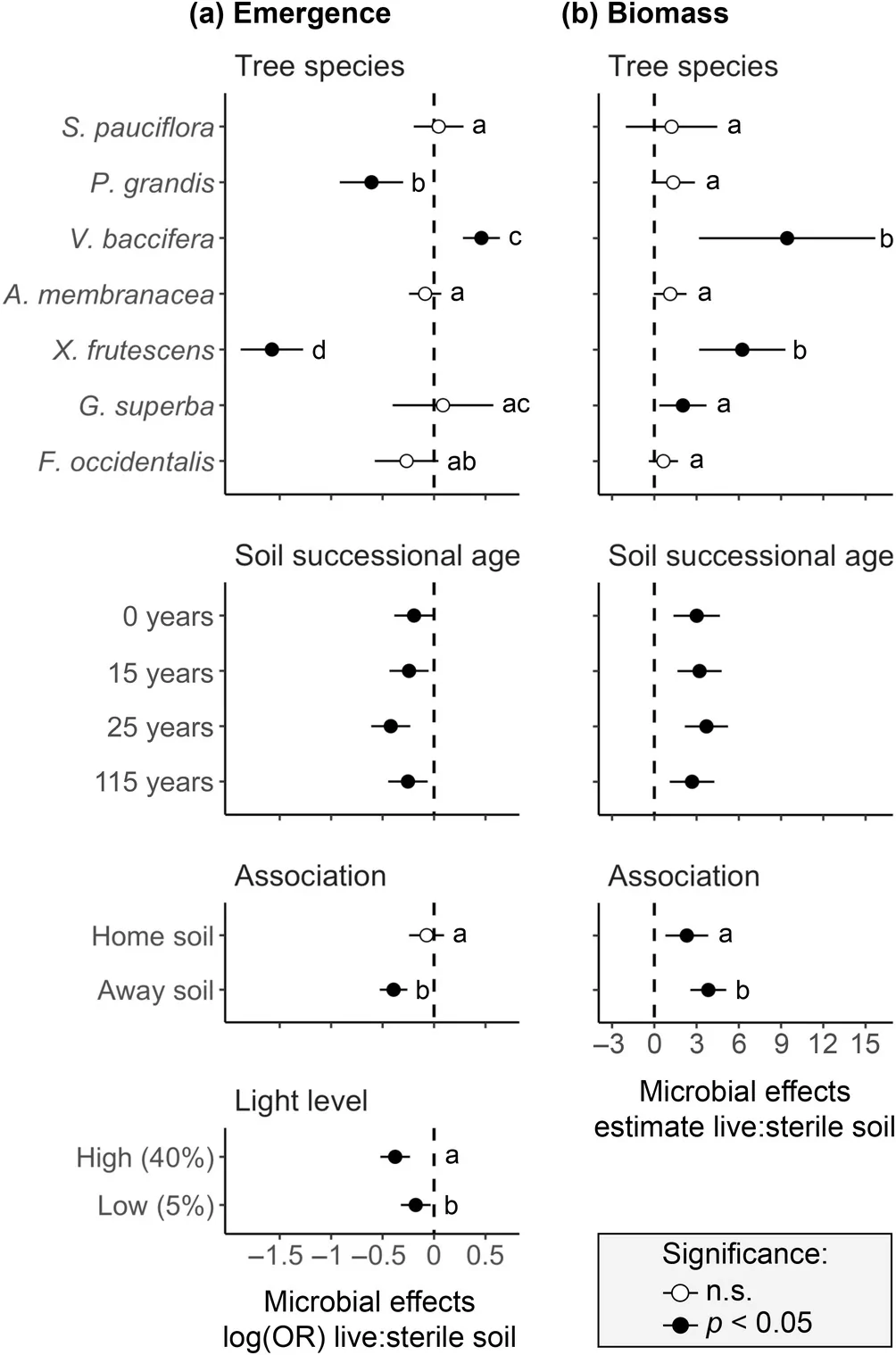
Microbial effects on (a) seedling emergence and (b) biomass. Microbial effects are shown for seven tree species (early‐ to late‐successional species sorted from top to bottom) in soils from four successional stages, in tree species' home versus away stages, and under high‐ and low‐light levels. Positive effects >0 indicate that (a) more seedlings emerged in live than sterilized soil and that (b) the predicted mean biomass (measured in g) was higher in live than sterilized soil. Letters represent significant differences between effects within a panel (*p* < 0.05). For model results, microbial effects, and pairwise contrasts see Appendix S1: Tables S6, S3, and S4, respectively.

Out of 15,211 sown seeds 5969 (39.2%) seedlings emerged within 415 days. In all successional stages, net microbial effects on emergence were negative (Figure 2a; Appendix S1: Tables S2 and S3), that is, the likelihood of seedling emergence was lower in live than sterilized soil. This effect did not differ among stages (𝘟^2^ (df) = 3.59 (3), *p* = 0.309; Appendix S1: Tables S2 and S4). Yet, microbial effects differed with tree species' association (𝘟^2^ (df) = 10.19 (1), *p* < 0.001; Figure 2a; Appendix S1: Table S2) and were negative in away (log(OR) = −0.39, 95% CI = −0.53 to −0.26) but neutral in home soils (log(OR) = −0.07, 95% CI = −0.24 to 0.10; Appendix S1: Table S3). Thus, the likelihood of emergence in live versus sterilized soils was 3.5% lower in home soils (*P*_live_ = 48.3%, *P*_sterilized_ = 51.8%) and 19.4% lower in away soils (*P*_live_ = 40.3%, *P*_sterilized_ = 59.7%). This difference was significant, and seedlings were more likely to emerge in live soils from successional stages in which they are abundant than in soils from away stages (estimate = −0.32, 95% CI = −0.52 to −0.12; Appendix S1: Table S4). Tree species identity strongly affected the strength and direction of microbial effects (𝘟^2^ (df) = 141.1 (6), *p* < 0.001; Appendix S1: Table S2). Two species, *Psychotria grandis* Sw. and *Xylopia frutescens* Aubl. experienced negative effects, while effects were positive for *Vismia baccifera* (L.) Triana & Planch. (Appendix S1: Table S3). In the remaining four species, which included early‐, mid‐, and late‐successional species, emergence did not differ significantly between live and sterilized soils (Figure 2a; Appendix S1: Table S3). Microbial effects were more negative under high than low‐light level (estimate = −0.20, 95% CI = −0.38 to −0.02; Appendix S1: Table S4).

### Biomass growth

Harvested seedlings had an average dry biomass of 4.39 g with large variation among species, seedling ages, and treatments (Appendix S1: Table S5). Microbial effects on biomass growth did not differ with successional stage (𝘟^2^ (df) = 1.49 (3), *p* = 0.684; Appendix S1: Table S2) and were generally positive (Figure 2b; Appendix S1: Table S3). Microbiota increased biomass growth in both away and home stages (Appendix S1: Table S3). This positive effect was stronger in away than home soils (estimate = 1.53, 95% CI = 0.04–3.01; Appendix S1: Table S4). Microbial effects on biomass growth were positive for three species and neutral for four species (Appendix S1: Table S3). The positive effect was stronger for *V. baccifera* and *X. frutescens*, two mid‐successional species, than for the other early‐ and late‐successional species (Figure 2b; Appendix S1: Table S4).

### Mortality

**FIGURE 3:**
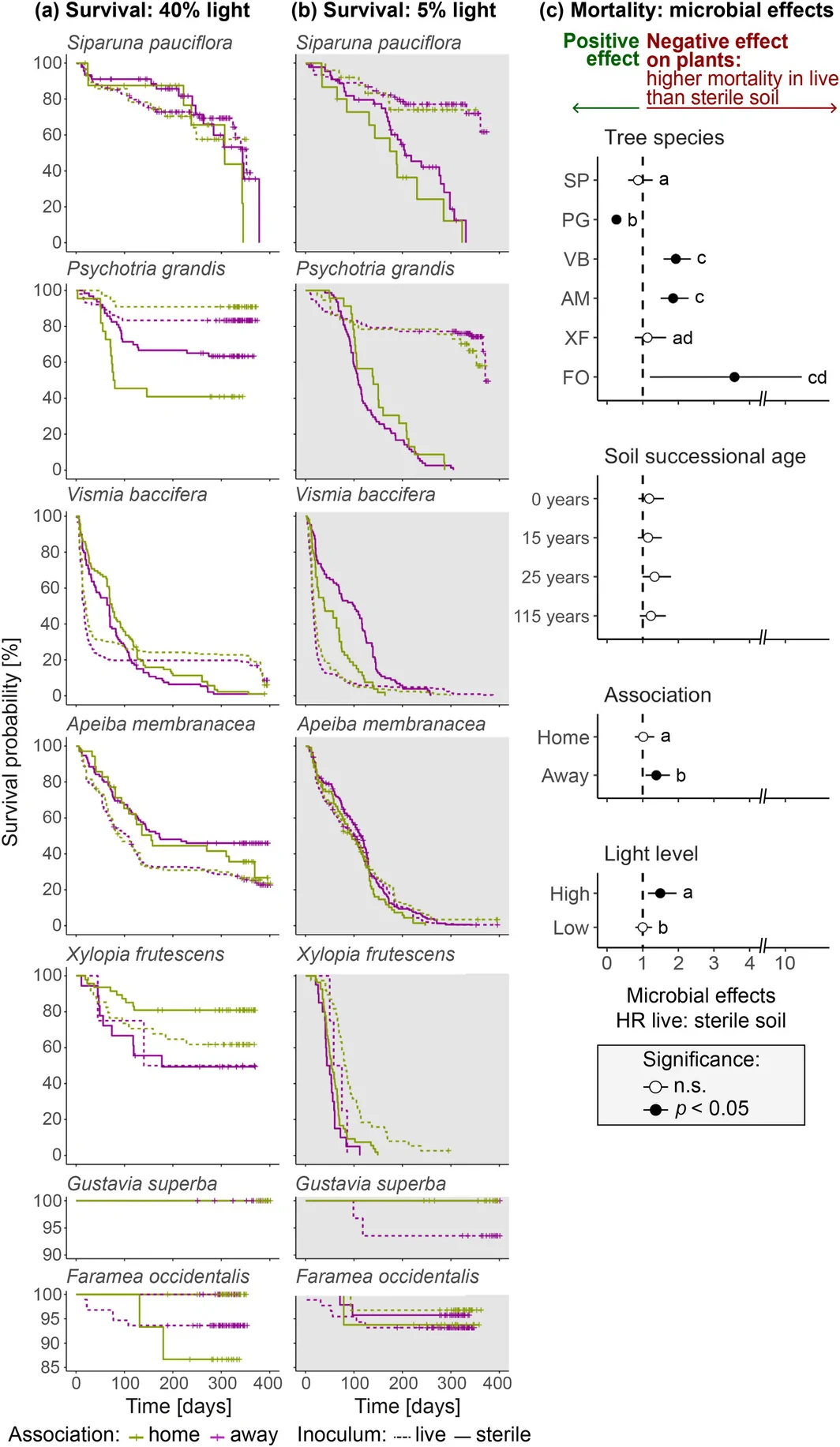
Seedling survival probabilities over 415 days for seven tree species in live and sterilized soils collected from four successional stages and pooled into species' home versus away stages under (a) 40% light and (b) 5% light. Additionally, (c) mortality hazard ratio (HR) is shown as the contrast between mortality in live versus sterilized soils for six tree species, in soils of four successional stages, and in tree species' home and away soils. A HR >1 indicates higher mortality in live than sterilized soil and thus a negative microbial effect on performance. Letters show differences between effects within each panel (*p* < 0.05). Contrasts could not be calculated for *Gustavia superba*, because all its seedlings in sterilized soils survived. For model results, microbial effects, and pairwise contrasts, see Appendix S1: Tables S6, S3, and S4, respectively.

Overall, 1263 seedlings (21.2% of emerged seedlings) survived over 415 days. Microbial effects on mortality did not vary with successional stage (𝘟^2^ (df) = 1.05 (3), *p* = 0.79; Appendix S1: Tables S2 and S4) and were neutral in all stages (Figure 3c; Appendix S1: Table S3). Soil microbiota from species' away stages increased mortality compared with species' home stages (estimate = 1.36, 95% CI = 1.07–1.74; Appendix S1: Table S4). Seedlings had a 38% higher mortality hazard in live than sterilized soil from away stages (HR = 1.38, 95% CI = 1.08–1.77), while there was no difference between live and sterilized soils from home stages (HR = 1.02, 95% CI = 0.78–1.33; Appendix S1: Table S3). Tree species identity strongly affected microbial effects (Appendix S1: Table S2) and microbiota significantly increased mortality for three species and decreased it for one species. Mortality was markedly lower in the two late‐successional species, only 1.2% for *G. superba* and 4.9% for *F. occidentalis*, than the average 65.8% mortality in the five species associated with early‐ and mid‐successional stages (range 36.9%–97.1%; Appendix S1: Table S5). However, microbial effects on mortality were not consistently stronger in the early‐successional species (Figure 3c; Appendix S1: Table S3). Microbial effects on mortality were more positive under high than low light (contrast estimate = 1.49, 95% CI = 1.19–1.88; Appendix S1: Tables S3 and S4), that is, microbiota increased mortality more under 40% than 5% daylight. The effect of light on mortality increased over time (coefficient = 0.01, 95% CI = 0.009–0.011), while that of microbiota decreased (coefficient = −0.007, 95% CI = −0.008 – −0.006; Appendix S1: Table S6).

## DISCUSSION

We analyzed seedling emergence, biomass growth, and mortality for 15,211 seeds of seven tree species in live soil versus sterilized and fungicide‐treated soil collected from forests of four successional stages to assess the role of microbial effects on tree species' establishment success during the first 115 years of tropical moist forest recovery. Microbial effects varied with tree species, tree species' association with soil successional stages, and light level, but not with soil successional stage itself. We found species‐specific net positive or net negative microbial effects on seedling performance in six of the seven tree species, highlighting the importance of microbial effects on seedling establishment and the impact of both mutualists and pathogens. Microbial effects were less negative on emergence and more positive on mortality in home soils, that is, soils from the successional stages in which a species is abundant, than in away soils. The effect of light on mortality increased over time, while that of microbiota decreased, indicating a shift in the determinants of mortality during the first year of seedling life. In summary, our results suggest that microbial effects are prevalent, specific to tree species and light levels, and that positive microbial effects may affect secondary forest succession by improving seedling performance at tree species' home stages.

### No variation in microbial effects with successional stage

Contrary to our hypothesis, microbial effects did not vary with soil successional stage. A recent study on soil microbial communities of nearby forests found that the diversity of both pathogenic and mycorrhizal fungi increased from pastures to younger to older secondary forests (Saltonstall et al., 2025). At the plant community level, the effects of antagonistic and mutualistic microbiota may therefore counterbalance one another, leading to no detectable change in net microbial effects with successional stage across tree species.

### Positive microbial home effects could influence forest succession

Tree species' association with successional stages affected microbial effects on all three performance measures (Figures 2 and 3). We expected stronger negative microbial effects in soils from home successional stages in which species are most abundant due to an accumulation of pathogens that are assumed to have higher host specificity than mutualists (Schroeder et al., 2019). While seedlings did have a lower biomass in live home than away soils (Figure 2), this effect was largely driven by one species, *P. grandis* (Appendix S2). In contrast to our expectation, microbiota reduced emergence to a much lower extent in species' home than away soils. Seedlings were also less likely to die if they grew in live home than away soils (Appendix S1: Table S3). Similarly positive plant–microbe interactions at home stages have been observed in grasslands (Kardol et al., 2006) and in some tree species (Pizano et al., 2014). The overall approximately 16% net higher emergence and approximately 36% lower mortality in live home than away soils that we found may substantially increase species' establishment success given the high overall seedling mortality of 70%–90% in Panamanian moist forests (Augspurger, 1984) and may promote species' persistence at home stages through continued establishment.

Positive microbial effects have been suggested to influence plant communities in various ecosystems (Tedersoo et al., 2020), to promote tree species' dominance in Bornean rainforests (Segnitz et al., 2020), and to slow secondary succession in grasslands (van der Putten et al., 2013). However, if plant–microbe interactions are altered by abiotic conditions specific to a successional stage, for example, light (Pizano et al., 2014), soil fertility (Segnitz et al., 2020), soil water content (Zhao et al., 2017), or by increased nutrient transfer from mycorrhizas to plants that provide more carbohydrates (Kiers et al., 2011), positive home effects may fade as succession progresses. It thus remains to be tested whether the positive microbial home effects that we found can counteract the competitive dominance of later‐successional species and contribute to the observed slow recovery of tree community composition in tropical forests (Poorter et al., 2021; Rozendaal et al., 2019). Nonetheless, our results suggest that tree species' association rather than successional stage per se is a key determinant of microbial effects. By enhancing seedling performance of individual species at their home stages, positive microbial effects may reinforce successional niches, promote species' dominance at their home stages, and could slow successional turnover of tree species.

It is important to note that we measured net microbial effects, which reflect the balance of pathogen‐mediated negative and mutualist‐mediated positive effects. Our findings may thus result from an absence of species‐specific pathogens, for example, due to these pathogens accumulating with a lag phase, or from an increased importance of species‐specific mutualists in home soils. While pathogen‐mediated negative and host species‐specific effects on seedlings are well‐documented (Eck et al., 2019; Spear & Broders, 2021) mutualists have often been considered to be less host‐specific (Schroeder et al., 2019; van der Putten et al., 2013). However, microbes may also indirectly affect seedling performance by mediating soil nutrient availability, and effective specialization of soil fungi can enhance seed viability (Sarmiento et al., 2017). Moreover, plant species identity affects mycorrhizal community composition (Mangan et al., 2010), suggesting that (effects of) mutualistic microbes may be more species‐specific than previously assumed.

### Early‐ versus late‐successional species

Seedling performance of all but one of the seven species included in our study was affected by microbial effects, suggesting that soil microbiota generally play an important role during the first year of a seedling's life. Microbial effects differed greatly among the species, with net positive, negative, and neutral effects. We found seedling mortality of the two late‐successional species to be extremely low at 1.8% in *G. superba* and 4.9% in *F. occidentalis*, and more than an order of magnitude lower than the 65.8% average mortality of the earlier successional species and the average seedling mortality of 70%–90% in Panamanian forests (Appendix S1: Table S5; Augspurger, 1984). The almost complete absence of seedling deaths and the lack of variation in mortality between live and sterilized soils suggest that mortality of these late‐successional species was largely independent of soil microbiota and aligns with the growth‐defense trade‐off (Coley & Barone, 1996) and previous studies showing that shade‐tolerant tree species are less susceptible to fungal pathogens (Spear & Broders, 2021) and experience more positive microbial effects than light‐demanding species (McCarthy‐Neumann & Kobe, 2008; Pizano et al., 2014). In our study, however, we found no consistent pattern of more negative microbial effects in light‐demanding, early‐successional than in shade‐tolerant, late‐successional species on emergence or growth, in contrast to earlier studies (e.g., Pizano et al., 2014). The small set of species in each successional group, together with high variability in microbial effects within groups (Appendix S2), may have obscured potential general effects. Overall, our results suggest that microbial effects on seedling performance are highly species‐specific, even among species associated with similar successional stages.

### Light availability alters microbial effects on seedling emergence and mortality

We found more negative microbial effects on seedling performance under high‐ than low‐light levels (Figures 1 and 2c). This result contrasts with our hypothesis and a previous study that found high light availability to outweigh the negative microbial effects on light‐demanding trees under lower light levels (Pizano et al., 2014). Sporulation, the relative abundance of host‐specific mycorrhizal fungi (Mangan et al., 2010), and carbon transfer from plants to mycorrhizal fungi (Kiers et al., 2011) are all higher under high light. Two factors could have contributed to our result. First, it is possible that our low‐light level did not reduce light sufficiently to induce a negative carbon balance in the seedlings. Our low‐light level (5% of daylight) provided more light than the 1%–2% typically available in the understory of mature tropical rainforests. Second, the necessity to limit our study to a small subset of species in this highly diverse ecosystem, albeit a large number compared with other fully factorial experiments, could have affected our results. A recent study showed interactive effects of tree life history strategy and light on plant–microbe interactions (Xi et al., 2023). Similarly, we found large variation among species in their sensitivity to microbial effects under both high‐ and low‐light levels, which was further influenced by whether the seedlings were grown in their home versus away soils (Figure 2; Appendix S2). Our data hence indicate that light level interacts with tree species identity and association to create highly context‐dependent microbial effects.

The influence of light on mortality increased, while that of microbial effects decreased, with seedling age (Appendix S1: Table S2). Young tropical tree seedlings are highly susceptible to microbial effects (Mangan et al., 2010; Sarmiento et al., 2017) and can be more prone to die from fungal infection than older seedlings (Simamora et al., 2017). In contrast, light limitation and dependence on mutualists may become more important for larger seedlings once seed reserves have been depleted and respiration costs increase. Because light availability in our experiment exceeded that of mature forest understories, it is possible that in natural forests, seedling growth is slowed down more and microbial effects remain relatively more important in regulating mortality than light for a longer period than observed in our experiment (e.g., Augspurger, 1984). This balance may rapidly change if canopy gaps form and seedlings respond with increased performance, making light the dominant determinant of mortality. Our results suggest a shift in the relative importance of microbial effects and light in determining seedling mortality during early seedling establishment, highlighting the need for long‐term studies to assess the importance of microbial effects for seedling establishment in natural forest settings.

Experimental shade house studies enable an in‐depth understanding of the effect of specific variables by removing other, potentially conflicting or enhancing variables, and are thus of particular importance for advancing basic science in complex environments. However, this simplification that benefits our understanding of the interplay between the selected variables comes at the cost of reducing environmental heterogeneity (Kivlin, 2018). In combination with the necessary assumptions of our chronosequence approach and the required methods of soil handling, we acknowledge that this may limit the generality of our findings (Segnitz et al., 2020). For instance, soil pooling enabled us to compare effects of the microbial communities typical of each successional stage yet eliminated within‐stage variation. Fungicide application was needed to ensure that sterilized soils remained microbe‐free during our experiment, yet may have affected nontarget, endophytic fungi and soil chemistry (Wang et al., 2025). Nonetheless, our results of negative and positive differences between sterilized and live soil within species suggest that the main effect of sterilization and fungicide was driven by the intended reduction in fungi. Lastly, our species‐level analyses revealed substantial interspecific differences in microbial effects (Appendix S2). Together with the small number of species per successional group and constraints on species selection (e.g., due to seed availability), this may influence how microbial effects scale to community‐level dynamics. Thus, further work is needed to test the generality of our results, identify responsible microbiota (e.g., using molecular methods), disentangle mutualist‐ versus pathogen‐mediated effects (including indirect effects via soil nutrients), and assess the context‐dependency of plant–microbe interactions (Xi et al., 2023). Shade house experiments including additional abiotic factors (e.g., soil moisture, air humidity) and field studies along successional gradients in temperate and tropical forests (e.g., comparing seedlings in untreated vs. fungicide‐sprayed plots) will be essential to evaluate the in situ importance of microbial effects.

### Importance of microbial studies for forest restoration

Our study contributes to understanding the role of soil microbiota in plant community assembly during secondary succession and thereby to establishing the knowledge base for microbial inoculation as an effective forest restoration tool. If positive microbial home effects are widespread, transplants of soil microbial communities from a species' home successional stage could enhance seedling establishment of early‐successional species to jump‐start recovery, or mid‐ to late‐successional species to support targeted functional recovery. However, such approaches may also reinforce successional niches and potentially slow subsequent compositional turnover. This highlights the importance of clearly defining restoration goals and careful consideration of the use of microbial inoculation. Given the central role of microbiota in carbon and nutrient cycling and plant drought and pathogen resistance (Averill et al., 2022; Baldrian et al., 2023), their importance for maintaining and restoring the functionality of forests globally will likely increase under accelerating land conversion, soil erosion, and climate change.

## CONCLUSION

We show that soil microbial effects influence tree species establishment during the secondary succession of species‐rich forests. Specifically, our study advances the understanding of how soil microbiota influence early life stages by demonstrating strong, species‐specific microbial effects on seedling emergence, growth, and mortality. The positive, microbial‐mediated home effects that we find suggest that microbiota may promote seedling performance at species' home successional stages and may thereby slow successional tree species turnover and have important implications for the development of microbial inoculation as a potentially powerful tool in forest restoration. Together with the interaction found between microbial effects and light, these results suggest that microbial effects are highly context‐dependent, enhance tree species niche differentiation, and accelerate environmental filtering of tree communities and thereby contribute to plant community assembly during forest recovery.

## AUTHOR CONTRIBUTIONS

All authors developed the study idea and design. AW conducted experiments, analyzed the data, and led the writing process. All authors contributed to the manuscript and gave their final approval.

## CONFLICT OF INTEREST STATEMENT

The authors declare no conflicts of interest.

## Supporting information


Appendix S1:

Appendix S2:

## Data Availability

Data (Weissflog et al., 2026) are available in Zenodo at https://doi.org/10.5281/zenodo.20701325.
