# Supplementary material for: Soil microbial effects on tree seedling performance during forest secondary succession
Source: Ecology. 2026 Sep 4;107(9):e70477. doi: 10.1002/ecy.70477 (PMC13543196; doi:10.1002/ecy.70477)
Supplement: Supplementary file 1 [file ECY-107-e70477-s001.pdf]

Appendix S1

Soil microbial effects on tree seedling performance during forest secondary succession

Anita Weissflog, Bettina M.J. Engelbrecht, John R. Healey, Lars Markesteijn

Ecology

Section S1

Experimental set-up. The shade house is located at ~70 m distance from a mature rainforest, has a concrete floor, a plastic roof and is covered entirely in shade-cloth allowing for airflow. Each 5 l pot (ø 21 cm) had five drainage holes and was placed on a 1 cm tall plastic ring at a distance of ≥ 5 cm from other pots. Metal grid tables promoted drainage and minimized the risk of microbial contamination among pots. We thoroughly sterilized all tables and working materials with household bleach before the experiment started and throughout the experiment where necessary.

To minimize bias from different quantities of water leaking and nutrient leaching, we made a single application of the slow-release fertilizer Osmocote Plus (N15–P9–K12; 18.55 g per pot; Scotts Co. LLC, Marysville, USA) in November 2019, i.e. 21, 10, and 5 months after sowing (due to staggered experimental start dates of species sets, see Table S1). Insect herbivory forced us to apply a broad-spectrum insecticide (Pyrethrin 1%, Naphtha 0.78%; Bonide LLC, Oriskany, USA) to all pots twice and to continuously remove all abscised leaves from experimental pots throughout the experiment.

Tree species information. All our species form associations with arbuscular mycorrhizas as documented at the species or genus level (Faramea occidentalis: Husband et al. 2002; Psychotria, Siparuna, Vismia: Kottke and Haug 2004; Apeiba membranacea: Lovelock et al. 2003; Gustavia superba: Pizano et al. 2017; Xylopia: Soudzilovskaia et al. 2022).

Interspecific variation in the timing of seed production and the rapid decline in the viability of seeds of many tropical tree species (Garwood 1983; Román et al. 2012) necessitated a staggered start to the experiment, with three species being sown in November 2018, two species in January 2019 and two species in June 2019. All seedlings were harvested in August 2020. Experimental duration was 631, 552 and 416 days respectively (Table S1). Species were sown at different times due to differences in their time of seed production and thus had different experimental durations (Table S1). We cut off emergence and mortality data after the study length for the species that were sown last minus one day, i.e. after 415 days, to equalize experimental time among species.

Species were sown at different times due to different seed production times (Table S1). Seeds were placed onto the inoculum layer (between 3 and 40 seeds per pot; Fig. 1). To limit competition, we thinned seedlings to a maximum of three seedlings per pot seven months after sowing. We thinned out a total of 1,889 seedlings seven months after the sowing date for each respective species set (Table S1).

Soil handling and pooling. Soil handling and storage has been suggested to contribute to reported differences in microbial communities between field sites and shade house pots in some studies (Kivlin 2018; but see: Pizano et al. 2014), yet storage at room temperature for 11 days did not alter bacterial abundance and communities in temperate forest soils (Brandt et al. 2014). We stored field soils for the shortest time possible given the complexity and scale of the experiment in a ventilated, dark room. All soils were used within 22 days after collection to inoculate the experimental pots either "live" (containing soil microbes) or "sterilized" (autoclaved for 2 x 57 min at 121 C; Sterilmatic, Market Forge Industries Inc., Everett, USA).

Our sampling aimed at encompassing small-scale variation in microbial communities (Schroeder et al. 2019). Soil pooling was the preferable method to compare effects of the microbial communities that are typical of each of our successional stages rather than assess within-stage variation. A meta-analysis found no bias from soil pooling on PSF strength or direction (Allen et al. 2021).

We applied live and sterilized forest soil inoculum at around 10% of pot volume. Using a small amount of inoculum minimizes potential bias from successional changes in soil pH and nutrients and nutrient pulses through microbial lysis after sterilization, while providing an effective microbial inoculum (Mangan et al. 2010).

Seeds were placed directly onto the inoculum layer before we sprinkled the entire surface with a ~1 mm thin layer of sterilized sand to reduce mold development.

Statistical analyses. We excluded one *Faramea occidentalis* pot (under 5% light with live soil) from the analysis as it was forgotten when sowing seeds.

Thinned out seedlings were not included in the mortality analysis.

We translated the logarithmic odds (log(OR)) ratios into probabilities (P) using the following formula: $P = 1/ 1+e^{\log(OR)}$.

All statistical analyses were conducted in R (R Core Team 2024). We used the R package 'lme4' (Bates et al. 2015) to conduct generalized linear mixed effects models on seedling emergence and linear mixed effects models on biomass growth. To analyze seedling mortality, we used the package 'coxme' (Therneau 2024b) to run Cox proportional mixed effects hazard models, the package 'survival' (Therneau 2024a) to test the underlying proportional hazard assumption, and the package 'Greg' (Gordon and Seifert 2022) to split our data into five 83-day intervals. We created Kaplan-Meier curves using the package 'survminer' (Kassambara et al. 2021). For all models, we tested for the normality of residuals, homoscedasticity, and non-multicollinearity using the 'performance' package (Lüdecke et al. 2021) and performed type III ANOVAs using the 'car' package (Fox and Weisberg 2019). Subsequently, we calculated microbial effects as the differences in model-predicted performance in live versus sterilized soil per species-treatment level and conducted Bonferroni-adjusted pairwise contrasts of microbial effects on seedling performance (i.e., separately for emergence, biomass growth, and mortality) between species, soil successional stage/association, and light levels using the 'emmeans' package (Lenth 2022).

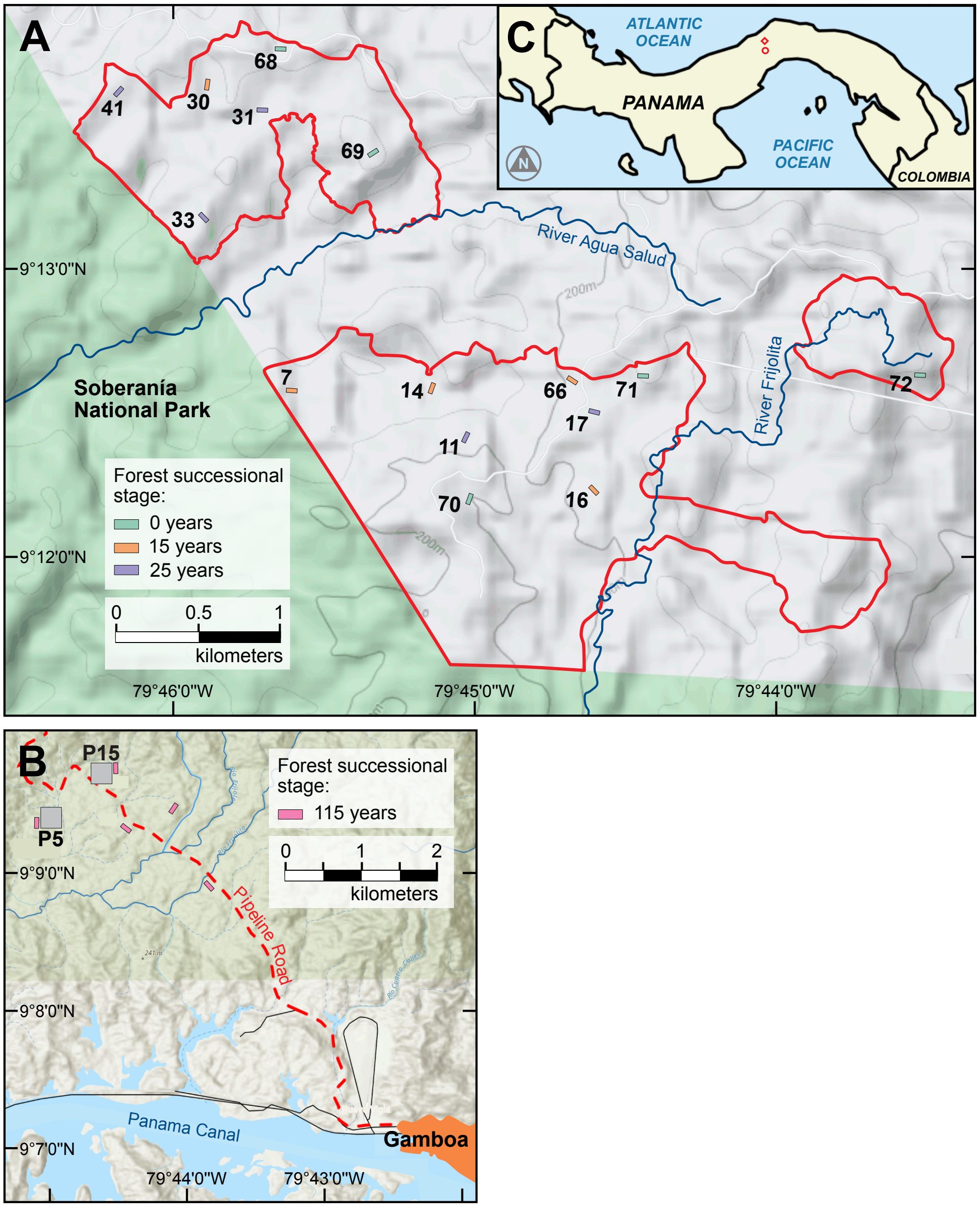

Figure S1. Location of field soil collection sites. We collected soils from five replicate sites for each of (**A**) three younger successional stages (0, 15 and 25 years of forest recovery) in the Agua Salud chronosequence and (**B**) one older successional stage (115 years) along Pipeline Road. The location of two ForestGeo long-term sampling sites (P5 and P15) is shown to facilitate orientation. (**C**) A country map shows the location of the Agua Salud sites (diamond) and the Pipeline Road sites (circle) in Central Panama.

Maps are strongly modified from (**A**) Google and (**B**) the Smithsonian Tropical Research Institute GIS portal. The **(C)** country map was created in R (R Core Team 2024) using natural earth data (https://www.naturalearthdata.com/) under creative commons license CC0 1.0: https://creativecommons.org/publicdomain/zero/1.0/.

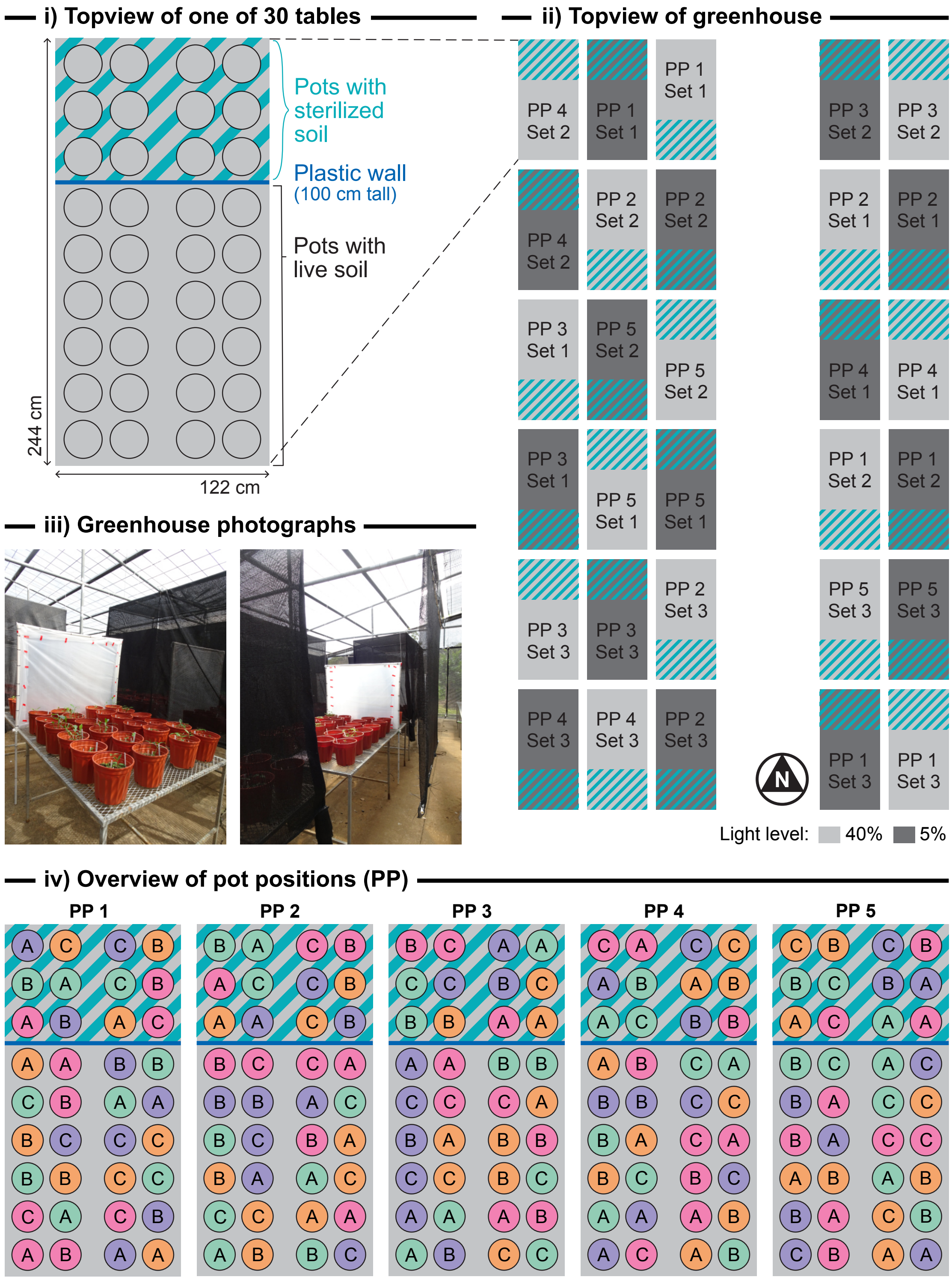

Species sets

Pot ID	Set 1	Set 2	Set 3
A	*Faramea occidentalis*	*Xylopia frutescens*	*Apeiba membranacea*
B	*Psychotria grandis*	*Vismia baccifera*	*Gustavia superba*
C	*Siparuna pauciflora*		

Soil successional stage:
0 yrs 15 yrs
25 yrs 115 yrs

Figure S2. Overview of experimental set-up in the shade house. Shown is a **i) Topview of one of 30 experimental tables**, on which 36 experimental pots (circles) were placed. Each table was divided into a section of pots filled with sterilized soil (turquoise shaded area) and a section of pots filled with live (i.e. including microbes) soil. These sections were separated by a 100 cm tall plastic wall (dark blue line). **ii) Topview of the shade house layout** showing the arrangement of the thirty experimental tables in the shade house, systematically alternating between tables exposed to the high-light (40% ambient light, pale grey) and tables exposed to the low-light (5% ambient light, dark grey) treatment. Individual table IDs provide information about the arrangement of pots (pot position: PP 1-5) and the identity of tree seedling species (Set 1-3) grown on each table. **iii) Photographs of experimental tables** in the shade house showing experimental pots, the separating plastic wall, and shade-cloth used to create the two different light treatments. **iv) Overview of the arrangement of experiment pots** on the tables. The experiment included seedlings of seven tree species, which were divided into three species sets (bottom left table). Seedlings were grown in soils from four successional stages (0, 15, 25 and 115 years of recovery) as indicated by different colours (bottom right legend). For each species x successional stage combination, we had ten pot replicates with live soil and five pot replicates with sterilized soil for each of the high-light and the low-light treatments. We created a template of pot positions randomizing species x successional stage treatments on five tables (with the only restriction being a maximum of two identical species x treatment combinations per table) shown as PP1-5. This template was then applied to both the five high-light and the five low-light tables of each species set (Set 1-3; see ii). All photographs by Anita Weissflog.

Table S1. Background information for seven tropical tree species. Species were selected based on their relative abundance in four successional stages (0, 15, 25 and 115 years of forest recovery). A species was associated with a successional stage, if it was among the 20 most abundant species in that successional stage. We calculated relative abundance as the importance value IV (= relative density plus relative basal area divided by two). IV can range from 0-1 with a higher value indicating higher abundance of a species in a respective successional stage. Seeds were collected from ≥ 6 adult trees per species ("n trees"). Dates of seed collection, soil collection, and thinning are shown. Due to interspecific variation in the timing of seed production, the experimental start date was staggered: species in set 1 were sown on 06.01.18 (DD.MM.YY) and harvested during 29.07.20-26.08.20, while experimental duration was 24.01.19-26.01.19 to 30.07.20-19.08.20 for set 2, and 14.06.19 to 03.08.20-07.08.20 for set 3, respectively.

Set	Species	Family	IV per successional stage				n trees	Dates (DD.MM.YY)		
			0	15	25	115		Seed collection	Soil collection	Thinning
1	*Siparuna pauciflora* (Beurl.) A.DC.	Monimiaceae	0.04				8	17.10. – 29.10.18	29.10. – 01.11.18	07.06. – 08.06.19
1	*Psychotria grandis* Sw.	Rubiaceae	0.02				18	18.10. – 01.11.18	29.10.- 01.11.18	07.06. – 08.06.19
2	*Vismia baccifera* (L.) Triana & Planch.	Hypericaceae	0.10	0.03			12	30.12.18 – 15.01.19	10.01.- 15.01.19	13.09.19
3	*Apeiba membranacea* Spruce ex Benth.	Tiliaceae		0.03			6	01.02.- 28.02.19	23.05.- 31.05.19	21.01.20
2	*Xylopia frutescens* Aubl.	Annonaceae	0.07	0.11	0.11		> 20	30.12.18 – 15.01.19	10.01.- 15.01.19	13.09.19
3	*Gustavia superba* (Kunth) O.Berg	Lecythidiaceae				0.06	19	15.05.– 14.06.19	23.05.- 31.05.19	21.01.20
1	*Faramea occidentalis* (L.) A.Rich.	Rubiaceae				0.91	40	15.10. – 30.10.18	29.10. – 01.11.18	07.06. – 08.06.19

Table S2. Results of type-III ANOVAs. Seeds were germinated in soils including a living microbial community or sterilized soils ("Inoculum" treatment) from forests of four stages of forest recovery ("Successional stage") under two light levels ("Light"). In separate analyses, we translated the successional stages into the binary variable "Association" that differentiates between home stages where a species is abundant and away stages. We show results for models on **successional stage** (left side of table) and **tree species' association** (right side of table) for **(a)** binomial generalized linear mixed effects models on emergence, **(b)** linear mixed effects models on biomass growth, and **(c)** cox-proportional hazards models on mortality.

**Successional stage**				**Association (home versus away)**			
**(a) Emergence**	χ^2	**df**	***p***		χ^2	**df**	***p***
Inoculum	0.73	1	0.394	Inoculum	7.77	1	**0.005**
Successional stage	7.97	3	**0.047**	Association	5.60	1	**0.018**
Light	1.38	1	0.241	Light	1.39	1	0.238
Species	489.61	6	**< 0.001**	Species	457.26	6	**< 0.001**
Inoculum x Succ. stage	3.59	3	0.309	Inoculum x Association	10.19	1	**0.001**
Inoculum x Light	4.86	1	**0.028**	Inoculum x Light	4.87	1	**0.027**
Inoculum x Species	141.10	6	**< 0.001**	Inoculum x Species	147.25	6	**< 0.001**
**(b) Biomass growth**	χ^2	**df**	***p***		χ^2	**df**	***p***
Inoculum	1.52	1	0.217	Inoculum	6.16	1	**0.013**
Successional Stage	0.14	3	0.987	Association	0.15	1	0.694
Species	111.99	6	**<0.001**	Species	113.77	6	**<0.001**
Seedling Age	47.38	1	**<0.001**	Seedling Age	48.38	1	**<0.001**
Seedlings per pot (harvest)	21.16	1	**<0.001**	Seedlings per pot (harvest)	19.74	1	**<0.001**
Inoculum x Succ. stage	1.49	3	0.684	Inoculum x Association	4.09	1	**0.043**
Inoculum x Species	18.69	6	**0.005**	Inoculum x Species	24.10	6	**<0.001**
**(c) Mortality**	χ^2	**df**	***p***		χ^2	**df**	***p***
Inoculum	22.58	1	**< 0.001**	Inoculum	44.99	1	**< 0.001**
Successional Stage	4.66	3	0.199	Association	3.73	1	0.053
Light	15.89	1	**< 0.001**	Light	16.92	1	**< 0.001**
Species	175.92	5	**< 0.001**	Species	167.76	5	**< 0.001**
Time x Inoculum	154.27	1	**< 0.001**	Time x Inoculum	155.79	1	**< 0.001**
Time x Light	392.08	1	**< 0.001**	Time x Light	392.50	1	**< 0.001**
Seedlings emerged per pot	2.67	1	0.103	Seedlings emerged per pot	2.42	1	0.119
Inoculum x Succ. stage	1.05	3	0.790	Inoculum x Association	6.19	1	**0.013**
Inoculum x Light	12.02	1	**< 0.001**	Inoculum x Light	13.04	1	**< 0.001**
Inoculum x Species	116.90	5	**< 0.001**	Inoculum x Species	120.42	5	**< 0.001**

Note: Statistically significant values ($p < 0.05$) indicated in bold. Shown are Chi-square values (χ^2), degrees of freedom (df) and p-values.

Table S3. Microbial effects on the performance of seven tropical tree species. Microbial effects are measured as the contrast in performance between live and sterilized soils for **(a) emergence** (logarithmic odds ratio, log(OR)) and **(b) biomass growth** (estimate), and **(c) mortality** (hazard ratio, HR) with the lower and upper 95% confidence intervals (LCL and UCL, respectively). We germinated and grew seedlings in live and sterilized soils from four successional ages, which can be categorized as either home (statistically pooling all successional stages in which a respective species is abundant) or away stages, and under 40% and 5% of ambient daylight.

	**(a) Emergence**			**(b) Biomass growth**			**(c) Mortality**		
**Species**	**log(OR)**	**LCL**	**UCL**	**estimate**	**LCL**	**UCL**	**HR**	**LCL**	**UCL**
S. pauciflora	0.05	-0.197	0.287	1.23	-2.026	4.476	0.87	0.590	1.282
P. grandis	-0.61	**-0.916**	**-0.300**	1.34	-0.204	2.889	0.26	**0.185**	**0.376**
V. baccifera	0.46	**0.281**	**0.640**	9.43	**3.166**	**15.700**	1.93	**1.584**	**2.351**
A. membranacea	-0.09	-0.244	0.071	1.13	-0.032	2.292	1.85	**1.499**	**2.292**
X. frutescens	-1.58	**-1.880**	**-1.271**	6.24	**3.175**	**9.315**	1.13	0.776	1.659
G. superba	0.09	-0.403	0.577	2.03	**0.354**	**3.705**	–	–	–
F. occidentalis	-0.27	-0.577	0.042	0.65	-0.397	1.690	3.58	**1.241**	**10.309**
**Successional Stage**	**log(OR)**	**LCL**	**UCL**	**estimate**	**LCL**	**UCL**	**HR**	**LCL**	**UCL**
0 years	-0.19	**-0.386**	**-0.003**	3.01	**1.360**	**4.661**	1.18	0.878	1.592
15 years	-0.24	**-0.433**	**-0.052**	3.21	**1.637**	**4.783**	1.15	0.859	1.537
25 years	-0.42	**-0.611**	**-0.230**	3.70	**2.163**	**5.240**	1.33	0.993	1.795
115 years	-0.25	**-0.446**	**-0.061**	2.68	**1.091**	**4.267**	1.23	0.917	1.651
**Association**	**log(OR)**	**LCL**	**UCL**	**estimate**	**LCL**	**UCL**	**HR**	**LCL**	**UCL**
home	-0.07	-0.243	0.098	2.31	**0.783**	**3.830**	1.02	0.776	1.329
away	-0.39	**-0.527**	**-0.259**	3.84	**2.556**	**5.114**	1.38	**1.080**	**1.768**
**Light level**	**log(OR)**	**LCL**	**UCL**	**estimate**	**LCL**	**UCL**	**HR**	**LCL**	**UCL**
40%	-0.38	**-0.522**	**-0.233**	–	–	–	1.49	**1.142**	**1.953**
5%	-0.18	**-0.323**	**-0.033**	–	–	–	1.00	0.789	1.267

Note: Log(OR) and estimates are significantly different from 0 (i.e., from an equal effect in live and sterile soil) if the 95% confidence interval does not include 0, HR is significantly different from 1 (i.e., from an equal risk in live and sterile soil) if the 95% CI confidence interval does not include 1. Such statistically significant values ($p < 0.05$) are printed in bold.

Table S4. Pairwise contrasts of microbial effects on seedling performance. We compared **(a)** the logarithmic odds ratio of **Emergence**, **(b)** mean predicted **Biomass growth,** and **(c)** the hazard ratio of **Mortality** in live versus sterilized soil among all seven tree species, among successional forest stages, between away and home soils, and between high- and low-light levels (first factor relative to second factor) using Bonferroni-adjusted pairwise contrasts (Lenth 2022). The species *G. superba* was not included in the mortality analysis because no seedlings of this species died in sterilized soils.

	**(a) Emergence**			**(b) Biomass growth**			**(c) Mortality**		
**Tree Species**	**contrast**	**LCL**	**UCL**	**contrast**	**LCL**	**UCL**	**contrast**	**LCL**	**UCL**
AM – FO	0.180	-0.167	0.528	0.48	-1.07	2.034	0.518	0.178	1.506
AM – GS	-0.173	-0.688	0.341	-0.90	-2.92	1.125	–	–	–
AM – PG	0.522	**0.175**	**0.868**	-0.21	-2.13	1.703	7.024	**4.734**	**10.423**
AM – SP	-0.131	-0.420	0.157	-0.10	-3.53	3.344	2.131	**1.396**	**3.252**
AM – VB	-0.547	**-0.786**	**-0.308**	-8.30	**-14.68**	**-1.926**	0.960	0.728	1.266
AM –XF	1.489	**1.147**	**1.831**	-5.12	**-8.38**	**-1.847**	1.633	**1.064**	**2.507**
FO – GS	-0.354	-0.933	0.226	-1.38	-3.36	0.590	–	–	–
FO – PG	0.341	-0.096	0.778	-0.70	-2.55	1.162	13.554	**4.497**	**40.853**
FO – SP	-0.312	-0.705	0.081	-0.58	-3.99	2.831	4.112	**1.354**	**12.489**
FO – VB	-0.727	**-1.085**	**-0.369**	-8.79	**-15.14**	**-2.429**	1.853	0.635	5.413
FO – XF	1.309	**0.875**	**1.743**	-5.60	**-8.84**	**-2.362**	3.152	**1.031**	**9.640**
GS – PG	0.695	**0.116**	**1.274**	0.69	-1.58	2.952	–	–	–
GS – SP	0.042	-0.505	0.589	0.81	-2.84	4.445	–	–	–
GS – VB	-0.374	-0.896	0.148	-7.40	**-13.89**	**-0.916**	–	–	–
GS – XF	1.662	**1.086**	**2.239**	-4.22	**-7.70**	**-0.726**	–	–	–
PG – SP	-0.653	**-1.045**	**-0.261**	0.12	-3.43	3.661	0.303	**0.183**	**0.503**
PG – VB	-1.068	**-1.426**	**-0.711**	-8.09	**-14.54**	**-1.638**	0.137	**0.092**	**0.204**
PG – XF	0.968	**0.535**	**1.401**	-4.90	**-8.32**	**-1.481**	0.233	**0.140**	**0.386**
SP – VB	-0.416	**-0.717**	**-0.114**	-8.21	**-15.25**	**-1.166**	0.451	**0.294**	**0.691**
SP – XF	1.620	**1.232**	**2.009**	-5.02	**-9.47**	**-0.573**	0.767	0.449	1.308
VB – XF	2.036	**1.683**	**2.389**	3.19	-3.77	10.147	1.701	**1.108**	**2.610**
**Successional Stage**	**contrast**	**LCL**	**UCL**	**contrast**	**LCL**	**UCL**	**contrast**	**LCL**	**UCL**
0 – 15 yrs	0.048	-0.202	0.299	-0.200	-1.953	1.55	1.028	0.754	1.40
0 – 25 yrs	0.226	-0.024	0.476	-0.691	-2.383	1.00	0.885	0.646	1.21
0 – 115 yrs	0.059	-0.193	0.311	0.332	-1.438	2.10	0.960	0.702	1.32
15 – 25 yrs	0.178	-0.071	0.427	-0.491	-2.171	1.19	0.861	0.636	1.17
15 – 115 yrs	0.011	-0.240	0.261	0.532	-1.223	2.29	0.934	0.689	1.27
25 – 115 yrs	-0.167	-0.417	0.084	1.023	-0.678	2.72	1.085	0.798	1.48
**Association**	**contrast**	**LCL**	**UCL**	**contrast**	**LCL**	**UCL**	**contrast**	**LCL**	**UCL**
away – home	-0.321	**-0.517**	**-0.124**	1.53	**0.043**	**3.01**	1.361	**1.07**	**1.74**
**Light level**	**contrast**	**LCL**	**UCL**	**contrast**	**LCL**	**UCL**	**contrast**	**LCL**	**UCL**
40% – 5%	-0.200	**-0.378**	**-0.022**	–	–	–	1.494	**1.19**	**1.88**

Note: Statistically significant values ($p < 0.05$) are printed in bold (see note Table S3, here applied for the pairwise comparison of factor levels). Species abbreviations: FO = *Faramea occidentalis*, GS = *Gustavia superba*, PG = *Psychotria grandis*, SP = *Siparuna pauciflora*, VB = *Vismia baccifera*, XF = *Xylopia frutescens*.

Table S5. Overview of seedling performance of seven tropical tree species. In a fully factorial design, we germinated seeds and grew seedlings of seven tree species under two light levels ("40% light" and "5% light") in soils collected from forests of four successional stages (0, 15, 25, and 115 years of recovery) that either had been sterilized ("sterile") or contained a living microbial community ("live"). We classified all successional stages at which a species is naturally abundant as "home" and pooled the other successional stages into "away". We present summary data for each species across all successional stages and separately for home and away stages. (**a) Emergence** is the number of seedlings emerged relative to the number of seeds sown with percentage emerging shown in brackets. We show the odds ratio (OR), which divides the odds of emergence in live soil by the odds of emergence in sterilized soil. An OR > 1 indicates a higher emergence in live than sterilized soil and thus a positive microbial effect. **(b) Biomass growth** is the across-seedling mean of dry weight (roots, stems, and leaves; in grams) and the standard error. We show **(c) Survival** as the number of seedlings that survived the first 415 days after sowing relative to the sum of seedlings that died and survived in the same time span, and in brackets percentage survival. We provide risk ratios (RR) as the percentage survival in live versus sterilized soil. A RR > 1 signifies higher survival in live than sterilized soil (positive microbial effect).

	**(a) Emergence**							
	**40% light**			**5% light**			**Across light**	
	**sterile**	**live**	**OR**	**sterile**	**live**	**OR**	**total**	**OR**
Siparuna pauciflora	78/ 370 (21.1%)	157/ 740 (21.2%)	1.01	71/ 370 (19.2%)	151/ 740 (20.4%)	1.08	457/ 2220 (20.6%)	1.04
Home	21/ 95 (22.1%)	34/ 190 (17.9%)	0.77	16/ 95 (16.8%)	29/ 190 (15.3%)	0.89	100/ 570 (17.5%)	0.82
Away	57/ 275 (20.7%)	123/ 550 (22.4%)	1.10	55/ 275 (20.0%)	122/ 550 (22.2%)	1.14	357/ 1650 (21.6%)	1.12
Psychotria grandis	115/ 160 (71.9%)	183/ 320 (57.2%)	0.52	114/ 160 (71.3%)	189/ 320 (59.1%)	0.58	601/ 960 (62.6%)	0.55
Home	29/ 40 (72.5%)	46/ 80 (57.5%)	0.51	26/ 40 (65.0%)	56/ 80 (70.0%)	1.26	157/ 240 (65.4%)	0.80
Away	86/ 120 (71.7%)	137/ 240 (57.1%)	0.53	88/ 120 (73.3%)	133/ 240 (55.4%)	0.45	444/ 720 (61.7%)	0.49
Vismia baccifera	207/ 600 (34.5%)	519/ 1200 (43.3%)	1.45	156/ 600 (26.0%)	449/ 1200 (37.4%)	1.7	1331/ 3600 (37.0%)	1.56
Home	102/ 300 (34.0%)	273/ 600 (45.5%)	1.62	103/ 300 (34.3%)	207/ 600 (34.5%)	3.15	670/ 1800 (37.2%)	2.16
Away	105/ 300 (35.0%)	246/ 600 (41.0%)	1.29	53/ 300 (17.7%)	242/ 600 (40.3%)	1.01	661/ 1800 (36.7%)	1.14
Apeiba membranacea	413/ 840 (49.2%)	724/ 1600 (45.4%)	0.85	427/ 800 (53.4%)	890/ 1600 (55.6%)	1.09	2454/ 4840 (50.7%)	0.97
Home	105/ 240 (43.8%)	189/ 400 (47.3%)	1.15	111/ 200 (55.5%)	222/ 400 (55.5%)	1.00	627/ 1240 (50.6%)	1.10
Away	308/ 600 (51.3%)	535/ 1200 (44.6%)	0.76	316/ 600 (52.7%)	668/ 1200 (55.7%)	1.13	1827/ 3600 (50.8%)	0.93

Table S5 (continued)

(a) Emergence

	**40% light**			**5% light**			**Across light**	
	**sterile**	**live**	**OR**	**sterile**	**live**	**OR**	**total**	**OR**
Xylopia frutescens	83/ 360 (23.1%)	38/ 720 (5.3%)	0.19	75/ 360 (20.8%)	42/ 720 (5.8%)	0.24	238/ 2160 (11.0%)	0.21
Home	62/ 270 (23.0%)	34/ 540 (6.3%)	0.23	55/ 270 (20.4%)	38/ 540 (7.0%)	0.30	189/1620 (11.7%)	0.26
Away	21/ 90 (23.3%)	4/ 180 (2.2%)	0.07	20/ 90 (22.2%)	4/ 180 (2.2%)	0.08	49/ 540 (9.1%)	0.08
Gustavia superba	17/ 60 (28.3%)	36/ 120 (30.0%)	1.08	19/ 60 (31.7%)	40/ 120 (33.3%)	1.08	112/ 363 (30.9%)	1.06
Home	7/ 15 (46.7%)	12/ 33 (36.4%)	0.65	6/ 15 (40.0%)	9/ 30 (30.0%)	0.64	34/ 93 (36.6%)	0.65
Away	10/ 45 (22.2%)	24/ 90 (26.7%)	1.27	13/ 45 (28.9%)	31/ 90 (34.4%)	1.29	78/ 270 (28.9%)	1.28
Faramea occidentalis	138/ 180 (76.7%)	249/ 360 (69.2%)	0.68	135/ 180 (75.0%)	254/ 351 (72.4%)	0.87	776/ 1071 (72.5%)	0.77
Home	27/ 45 (60.0%)	54/ 90 (60.0%)	1.00	26/ 45 (57.8%)	78/ 90 (86.7%)	4.75	185/ 270 (68.5%)	1.92
Away	111/ 135 (82.2%)	195/ 270 (72.2%)	0.56	109/ 135 (80.7%)	176/ 261 (67.4%)	0.49	591/ 801 (73.8%)	0.53
**Across species**	1051/ 2570 (40.9%)	1906/ 5063 (37.7%)	0.87	997/ 2530 (39.4%)	2015/ 5051 (39.9%)	1.02	5969/15214 (39.2%)	0.94
Home	353/ 1005 (35.1%)	642/ 1933 (33.2%)	0.92	293/ 965 (30.4%)	674/ 1930 (34.9%)	1.23	1962/ 5833 (33.6%)	1.05
Away	698/ 1565 (44.6%)	1264/ 3130 (40.4%)	0.84	654/ 1565 (41.8%)	1341/ 3121 (42.9%)	0.92	4007/ 9381 (42.7%)	0.88

(b) Biomass growth

	**40% light**		**5% light**			
	**sterile**	**live**	**sterile**	**live**		
Siparuna pauciflora	1.0 ± 0.14	3.0 ± 0.73	–	0.2 ± 0.08	–	–
Psychotria grandis	3.9 ± 0.46	6.5 ± 0.64	–	0.2 ± 0.02	–	–
Vismia baccifera	1.0 ± 0.34	10.7 ± 1.77	–	–	–	–
Apeiba membranacea	5.8 ± 0.58	8.6 ± 0.72	0.01 ± 0.00	0.1 ± 0.02	–	–
Xylopia frutescens	2.3 ± 0.42	9.7 ± 1.51	–	–	–	–
Gustavia superba	10.6 ± 1.30	15.1 ± 1.54	3.9 ± 0.93	4.4 ± 0.46	–	–
Faramea occidentalis	1.9 ± 0.15	3.8 ± 0.26	0.3 ± 0.02	0.4 ± 0.02	–	–

Table S5 (continued)

	(c) Survival							
	40% light			5% light			Across light	
	**sterile**	**live**	**RR**	**sterile**	**live**	**RR**	**total**	**RR**
Siparuna pauciflora	40/ 62 (64.5%)	81/ 122 (66.4%)	1.03	17/ 59 (28.8%)	89/ 117 (76.1%)	2.64	227/ 360 (63.1%)	1.51
Home	10/ 17 (58.8%)	18/ 28 (64.3%)	1.09	3/ 15 (20.0%)	19/ 25 (76.0%)	3.80	50/ 85 (58.8%)	1.72
Away	30/ 45 (66.7%)	63/ 94 (67.0%)	1.01	14/ 44 (31.8%)	70/ 92 (76.1%)	2.39	177/ 275 64.4%	1.45
Psychotria grandis	49/ 85 (57.6%)	115/ 135 (85.2%)	1.48	0/ 101 (0.0%)	98/ 138 (71.0%)	–	262/ 459 (57.1%)	2.96
Home	9/ 22 (40.9%)	30/ 33 (90.9%)	2.22	0/ 23 (0.0%)	24/ 37 (64.9%)	–	63/ 115 (54.8%)	3.86
Away	40/ 63 (63.5%)	85/ 102 (83.3%)	1.31	0/ 78 (0.0%)	74/ 101 (73.3%)	–	199/ 344 (57.8%)	2.76
Vismia baccifera	4/ 191 (2.1%)	32/ 428 (7.5%)	3.57	0/ 155 (0.0%)	0/ 447 (0.0%)	1.00	36/ 1221 (2.9%)	3.16
Home	2/ 92 (2.2%)	13/ 215 (6.0%)	2.78	0/ 53 (0.0%)	0/ 242 (0.0%)	1.00	15/ 602 (2.5%)	2.06
Away	2/ 99 (2.0%)	19/ 213 (8.9%)	4.42	0/ 102 (0.0%)	0/ 205 (0.0%)	1.00	21/ 619 (3.4%)	4.57
Apeiba membranacea	56/ 130 (43.1%)	90/ 370 (24.3%)	0.56	26/ 270 (9.6%)	38/ 571 (6.7%)	0.69	210/ 1341 (15.7%)	0.66
Home	12/ 35 (34.3%)	25/ 97 (25.8%)	0.75	4/ 75 (5.3%)	12/ 133 (9.0%)	1.69	53/ 340 (15.6%)	1.11
Away	44/ 95 (46.3%)	65/ 273 (23.8%)	0.51	22/ 195 (11.3%)	26/ 438 (5.9%)	0.53	157/ 1001 (15.7%)	0.56
Xylopia frutescens	47/ 65 (72.3%)	23/ 38 (60.5%)	0.84	0/ 74 (0.0%)	1/ 42 (2.4%)	–	71/ 219 (32.4%)	0.89
Home	38/ 47 (80.9%)	21/ 34 (61.8%)	0.76	0/ 54 (0.0%)	1/ 38 (2.6%)	–	60/ 173 (34.7%)	0.81
Away	9/ 18 (50.0%)	2/ 4 (50.0%)	1.00	0/ 20 (0.0%)	0/ 4 (0.0%)	1.00	11/ 46 (23.9%)	1.06
Gustavia superba	17/ 17 (100%)	36/ 36 (100%)	1.00	19/ 19 (100%)	38/ 40 (95.0%)	0.95	110/ 112 (98.2%)	0.97
Home	7/ 7 (100.0%)	12/ 12 (100.0%)	1.00	6/ 6 (100.0%)	9/ 9 (100.0%)	1.00	34/ 34 (100.0%)	1.00
Away	10/ 10 (100.0%)	24/ 24 (100.0%)	1.00	13/ 13 (100.0%)	29/ 31 (93.5%)	0.94	76/ 78 (97.4%)	0.96
Faramea occidentalis	58/ 2 (96.7%)	117/ 6 (95.1%)	0.98	60/ 63 (95.2%)	112/ 119 (94.1%)	0.99	347/ 365 (95.1%)	0.99
Home	13/ 15 (86.7%)	29/ 29 (100.0%)	1.15	15/ 16 (93.8%)	30/ 31 (96.8%)	1.03	87/ 91 (95.6%)	1.09
Away	45/ 45 (100.0%)	88/ 94 (93.6%)	0.94	45/ 47 (95.7%)	82/ 88 (93.2%)	0.97	260/ 274 (94.9%)	0.95
**Across species**	271/ 610 (44.4%)	494/1252 (39.5%)	0.89	122/ 741 (16.5%)	376/1474 (25.5%)	1.55	1263/4077 (31.0%)	1.10
Home	91/ 235 (38.7%)	148/ 448 (33.0%)	0.85	28/ 242 (11.6%)	95/ 515 (18.4%)	1.59	362/1440 (25.1%)	1.07
Away	180/ 375 (48.0%)	346/ 804 (43.0%)	0.90	94/ 499 (18.8%)	281/ 959 (29.3%)	1.55	901/2637 (34.2%)	1.13

Table S6. Summary of linear mixed effects models on (a) seedling emergence and (b) biomass growth as seedling dry weight, and **cox proportional hazards models on (c) seedling mortality**. Seedling performance was modeled as a function of inoculum (live versus sterilized soils), soil successional stage (upper table section: 0, 15, 25, and 115 yrs of forest recovery) or association (lower table section: home versus away soils), and light level (40% versus 5% of daylight). We show coefficients and their lower and upper 95% confidence levels (LCL and UCL) for fixed effects and variance estimates, standard deviation (sd), and number of observations for random effects.

	**Soil Successional Stage**								
	**(a) Emergence**			**(b) Biomass growth**			**(c) Mortality**		
**Fixed effects**	**coeff.**	**LCL**	**UCL**	**coeff.**	**LCL**	**UCL**	**coeff.**	**LCL**	**UCL**
Intercept	0.05	-0.153	0.255	6.00	**0.986**	**11.016**	–	–	–
Inoculum: live	-0.10	-0.340	0.134	0.99	-0.582	2.564	0.78	**0.461**	**1.108**
Soil Stage: 15 yrs	0.14	-0.062	0.345	-0.19	-1.578	1.196	0.02	-0.231	0.264
Soil Stage: 25 yrs	0.25	**0.052**	**0.458**	-0.06	-1.384	1.260	-0.16	-0.414	0.089
Soil Stage: 115 yrs	0.02	-0.189	0.221	-0.23	-1.614	1.154	0.10	-0.153	0.348
Species: FO	1.07	**0.770**	**1.365**	-5.67	**-7.393**	**-3.946**	-4.01	**-4.947**	**-3.077**
Species: GS	-0.98	**-1.405**	**-0.559**	1.67	-0.125	3.469	–	–	–
Species: PG	0.84	**0.543**	**1.140**	-5.40	**-7.354**	**-3.453**	0.01	-0.325	0.325
Species: SP	-1.50	**-1.753**	**-1.255**	-6.82	**-9.182**	**-4.457**	-0.66	**-1.046**	**-0.269**
Species: VB	-0.97	**-1.186**	**-0.760**	-9.80	**-15.807**	**-3.784**	1.16	**0.871**	**1.443**
Species: XF	-1.41	**-1.654**	**-1.158**	-6.78	**-9.442**	**-4.121**	0.44	**0.081**	**0.797**
Light: low (5%)	-0.10	-0.260	0.065	–	–	–	0.51	**0.258**	**0.757**
Seedling Age	–	–	–	2.06	**1.473**	**2.645**	–	–	–
Seedlings in pot (harvest)	–	–	–	-0.89	**-1.271**	**-0.512**	–	–	–
Seedlings in pot (emerged)	–	–	–	–	–	–	0.08	-0.017	0.183
Time x Inoculum	–	–	–	–	–	–	-0.01	**-0.008**	**-0.006**
Time x Light	–	–	–	–	–	–	0.01	**0.009**	**0.012**
Inoculum x 15 yrs	-0.05	-0.299	0.202	0.20	-1.549	1.949	-0.03	-0.338	0.282
Inoculum x 25 yrs	-0.23	-0.476	0.024	0.69	-0.997	2.379	0.12	-0.193	0.437
Inoculum x 115 yrs	-0.06	-0.311	0.193	-0.33	-2.098	1.434	0.04	-0.274	0.354
Inoculum x FO	-0.18	-0.528	0.167	-0.48	-2.031	1.062	0.66	-0.409	1.723
Inoculum x GS	0.17	-0.341	0.688	0.90	-1.119	2.919	–	–	–
Inoculum x PG	-0.52	**-0.868**	**-0.175**	0.21	-1.697	2.122	-1.95	**-2.344**	**-1.555**
Inoculum x SP	0.13	-0.157	0.420	0.10	-3.332	3.522	-0.76	**-1.179**	**-0.334**
Inoculum x VB	0.55	**0.308**	**0.786**	8.30	**1.938**	**14.667**	0.04	-0.236	0.317
Inoculum x XF	-1.49	**-1.831**	**-1.147**	5.11	**1.856**	**8.372**	-0.49	**-0.919**	**-0.062**
Inoculum x Light	0.20	**0.022**	**0.378**	–	–	–	-0.40	**-0.629**	**-0.175**
**Random effects**	**Variance**	**sd**	**Nr. obs.**	**Variance**	**sd**	**Nr. obs.**	**Variance**	**sd**	
Pot ID	0.07	0.26	839	–	–	–	0.11	0.34	
Table	0.01	0.10	33	0.20	0.45	29	0.05	0.22	
Light	–	–	–	11.89	3.45	2	–	–	

Table S6 (continued)

	Association (home versus away soil)								
	(a) Emergence			(b) Biomass growth			(c) Mortality		
**Fixed effects**	**coeff.**	**LCL**	**UCL**	**coeff.**	**LCL**	**UCL**	**coeff.**	**LCL**	**UCL**
Intercept	0.20	**0.036**	**0.369**	5.84	**0.059**	**11.715**	–	–	–
Inoculum: live	-0.27	**-0.455**	**-0.079**	1.53	**0.326**	**2.712**	0.92	**0.650**	**1.187**
Association: home	-0.19	**-0.352**	**-0.033**	0.24	-0.942	1.444	0.19	-0.003	0.380
Species: FO	1.06	**0.770**	**1.365**	-5.68	**-7.509**	**-4.002**	-4.02	**-4.961**	**-3.088**
Species: GS	-0.98	**-1.405**	**-0.558**	1.64	-0.271	3.384	–	–	–
Species: PG	0.84	**0.542**	**1.139**	-5.41	**-7.510**	**-3.464**	-0.01	-0.333	0.321
Species: SP	-1.50	**-1.750**	**-1.252**	-6.80	**-9.212**	**-4.501**	-0.66	**-1.047**	**-0.266**
Species: VB	-0.92	**-1.138**	**-0.705**	-9.90	**-15.884**	**-4.049**	1.11	**0.819**	**1.399**
Species: XF	-1.31	**-1.568**	**-1.048**	-6.86	**-9.543**	**-4.227**	0.33	-0.036	0.706
Light: low (5%)	-0.10	-0.260	0.065	–	–	–	0.53	**0.275**	**0.777**
Seedling Age	–	–	–	2.07	**1.492**	**2.706**	–	–	–
Seedlings in pot (harvest)	–	–	–	-0.86	**-1.232**	**-0.482**	–	–	–
Seedlings in pot (emerged)	–	–	–	–	–	–	0.08	-0.021	0.179
Time x Inoculum	–	–	–	–	–	–	-0.01	**-0.008**	**-0.006**
Time x Light	–	–	–	–	–	–	0.01	**0.009**	**0.012**
Inoculum x Association	0.32	**0.124**	**0.517**	-1.53	**-3.014**	**-0.072**	-0.31	**-0.551**	**-0.654**
Inoculum x FO	-0.18	-0.528	0.168	-0.53	-2.046	0.100	0.66	-0.406	1.730
Inoculum x GS	0.17	-0.342	0.687	0.98	-0.990	3.004	–	–	–
Inoculum x PG	-0.52	**-0.866**	**-0.173**	0.11	-1.756	2.005	-1.96	**-2.353**	**-1.559**
Inoculum x SP	0.13	-0.159	0.419	0.33	-3.031	3.678	-0.77	**-1.196**	**-0.346**
Inoculum x VB	0.47	**0.221**	**0.710**	8.98	**2.739**	**15.286**	0.11	-0.176	0.391
Inoculum x XF	-1.65	**-2.007**	**-1.293**	6.29	**2.992**	**9.606**	-0.35	-0.797	0.107
Inoculum x low light	0.20	**0.022**	**0.378**	–	–	–	-0.42	**-0.651**	**-0.193**
**Random effects**	**Variance**	**sd**	**Nr. obs.**	**Variance**	**sd**	**Nr. obs.**	**Variance**	**sd**	
Pot ID	0.07	0.26	839	–	–	–	0.12	0.35	
Table	0.10	0.10	33	0.21	0.46	29	0.05	0.22	
Light	–	–	–	11.66	3.41	2	–	–	

Note: Model coefficients for which the 95% confidence interval does not include 0 contribute significantly (at $p < 0.05$) to explaining variation in seedling performance and are printed in bold. Species abbreviations: FO = *Faramea occidentalis*, GS = *Gustavia superba*, PG = *Psychotria grandis*, SP = *Siparuna pauciflora*, VB = *Vismia baccifera*, XF = *Xylopia frutescens*.

References

Allen, Warwick J., Sarah J. Sapsford, and Ian A. Dickie. 2021. "Soil Sample Pooling Generates No Consistent Inference Bias: A Meta-analysis of 71 Plant–Soil Feedback Experiments." *New Phytologist* 231 (4): 1308–15. https://doi.org/10.1111/nph.17455.

Bates, Douglas, Martin Maechler, Ben Bolker, and Steve Walker. 2015. "Fitting Linear Mixed-Effects Models Using Lme4." *Journal of Statistical Software* 67 (1): 1–48. https://doi.org/10.18637/jss.v067.i01.

Brandt, Franziska B., Björn Breidenbach, Kristof Brenzinger, and Ralf Conrad. 2014. "Impact of Short-Term Storage Temperature on Determination of Microbial Community Composition and Abundance in Aerated Forest Soil and Anoxic Pond Sediment Samples." *Systematic and Applied Microbiology* 37 (8): 570–77. https://doi.org/10.1016/j.syapm.2014.10.007.

Fox, John, and Sanford Weisberg. 2019. *An R Companion to Applied Regression*. 3rd ed. Sage. https://www.john-fox.ca/Companion/.

Garwood, Nancy C. 1983. "Seed Germination in a Seasonal Tropical Forest in Panama: A Community Study." *Ecological Monographs* 53 (2): 159–81.

Gordon, M., and R. Seifert. 2022. *Greg: Regression Helper Functions*. V. 1.4.1. Released. https://CRAN.R-project.org/package=Greg.

Husband, R., E. A. Herre, S. L. Turner, R. Gallery, and J. P. W. Young. 2002. "Molecular Diversity of Arbuscular Mycorrhizal Fungi and Patterns of Host Association over Time and Space in a Tropical Forest." *Molecular Ecology* 11 (12): 2669–78. https://doi.org/10.1046/j.1365-294X.2002.01647.x.

Kassambara, Alboukadel, Marcin Kosinski, and Przemyslaw Biecek. 2021. *Survminer: Drawing Survival Curves Using "Ggplot2".* V. version 0.4.9. Released. https://CRAN.R-project.org/package=survminer.

Kivlin, Stephanie N. 2018. "Heterogeneity in Arbuscular Mycorrhizal Fungal Communities May Contribute to Inconsistent Plant-Soil Feedback in a Neotropical Forest." *Plant Soil*, no. 432: 29–44. https://doi.org/10.1007/s11104-018-3777-4.

Kottke, Ingrid, and Ingeborg Haug. 2004. "The Significance of Mycorrhizal Diversity of Trees in the Tropical Mountain Forest of Southern Ecuador." *Lyonia* 7 (December): 49–56.

Lenth, R. 2022. *Emmeans: Estimated Marginal Means, Aka Least-Squares Means*. V. 1.8.1-1. Released. https://CRAN.R-project.org/package=emmeans.

Lovelock, Catherine E., Kelly Andersen, and Joseph B. Morton. 2003. "Arbuscular Mycorrhizal Communities in Tropical Forests Are Affected by Host Tree Species and Environment." *Oecologia* 135 (2): 268–79. https://doi.org/10.1007/s00442-002-1166-3.

Lüdecke, Daniel, Mattan S. Ben-Shachar, Indrajeet Patil, Philip Waggoner, and Dominique Makowski. 2021. "Performance: An R Package for Assessment, Comparison and Testing of Statistical Models." *Journal of Open Source Software* 6 (60): 3139. https://doi.org/10.21105/joss.03139.

Mangan, Scott A., Edward A. Herre, and James D. Bever. 2010. "Specificity between Neotropical Tree Seedlings and Their Fungal Mutualists Leads to Plant–Soil Feedback." *Ecology* 91 (9): 2594–603. https://doi.org/10.1890/09-0396.1.

Pizano, Camila, Scott A. Mangan, James H. Graham, and Kaoru Kitajima. 2014. "Habitat-Specific Positive and Negative Effects of Soil Biota on Seedling Growth in a Fragmented Tropical Montane Landscape." *Oikos* 123 (7): 846–56. https://doi.org/10.1111/oik.01032.

Pizano, Camila, Scott A. Mangan, James H. Graham, and Kaoru Kitajima. 2017. "Host-Specific Effects of Soil Microbial Filtrates Prevail over Those of Arbuscular Mycorrhizae in a Fragmented Landscape." *Ecological Applications* 27 (6): 1946–57. https://doi.org/10.1002/eap.1579.

R Core Team. 2024. *R: A Language and Environment for Statistical Computing*. R Foundation for Statistical Computing, released. https://www.R-project.org/.

Román, Francisco, Adriana Sautu, Jefferson S. Hall, et al. 2012. *Guía Para La Propagacion de 120 Árboles Nativos de Panama y El Neotropico*.

Schroeder, John W., Jessica T. Martin, Diego F. Angulo, et al. 2019. "Host Plant Phylogeny and Abundance Predict Root-associated Fungal Community Composition and Diversity of Mutualists and Pathogens." *Journal of Ecology* 107 (4): 1557–66. https://doi.org/10.1111/1365-2745.13166.

Soudzilovskaia, N. A., S. Vaessen, M. Barcelo, et al. 2022. "Taxon Occurrence Data for the FungalRoot Database." PlutoF. https://doi.org/10.15468/a7ujmj.

Therneau, TM. 2024a. *A Package for Survival Analysis in R*. V. 3.7-0. Released. https://CRAN.R-project.org/package=survival.

Therneau, TM. 2024b. *Coxme: Mixed Effects Cox Models*. V. 2.2-22. Released. https://CRAN.R-project.org/package=coxme.
