# Supplementary material for: Soil microbial effects on tree seedling performance during forest secondary succession
Source: Ecology. 2026 Sep 4;107(9):e70477. doi: 10.1002/ecy.70477 (PMC13543196; doi:10.1002/ecy.70477)
Supplement: Supplementary file 2 [file ECY-107-e70477-s002.pdf]

Appendix S2

Soil microbial effects on tree seedling performance during forest secondary succession

Anita Weissflog, Bettina M.J. Engelbrecht, John R. Healey, Lars Markesteijn

Ecology

Species-level analyses

In addition to the analyses presented in the main manuscript, we analysed the effects of soil microbiota (Inoculum: sterilized versus live), soil successional stage (Successional stage: 0, 15, 25, 115 yrs)/species association (Association: home versus away), and light level (Light: 40% versus 5%) on emergence, biomass growth, and survival of each of the seven tree species individually. While these analyses have the advantage of enabling a more in-depth understanding of underlying processes, they also require much more data and model adjustment, and the complexity of the within-species variation may obscure the larger and potentially more relevant patterns across species.

Emergence

We analysed the odds of emergence (emerged seedlings divided by seeds that did not emerge) using a binomial generalized linear model with logit link (function glm in package stats; R Core Team 2024) for each species in the successional stage/association analyses. In one species-treatment combination (*G. superba*, 40% light, sterilized, 15 yrs) no seedling emerged. To avoid unreliable estimates due to complete separation we fitted a binomial Bayesian generalized linear model with logit link, a prior scale of 2.5 and used the number of predictors as a prior for the degrees of freedom (bayesglm in arm; Gelman and Su 2022) for this species.

To test our hypotheses, we *a priori* defined inoculum, successional stage/association and light as main effects. Using backward stepwise selection we then chose the best fitting models according to the Bayesian information criterion (Burnham and Anderson 2004). Final models included the main effects, and their two-way and three-way interactions. Adding seedling position in the shade house as a random effect contributed less than 1% of the explained variance and was omitted. We found no collinearity among our factors (vif in performance; Lüdecke et al. 2021) and no overdispersion (residual deviance by degrees of freedom).

We ran type III ANOVAs (Anova in car; Fox and Weisberg 2019) to assess the overall impact of main effects and interaction terms. We calculated microbial effects as logarithmic odds ratios (log(OR)) of emergence in live versus sterilized soils for each species x treatment combination (emmeans; Lenth 2022). Log(OR) > 0 indicate positive microbial effects and higher emergence in live than sterilized soil. We tested whether microbial effects differ among successional stage/ association with Bonferroni-adjusted pairwise contrasts within each species x light combination and averaged across light levels (contrasts in emmeans; Lenth 2022).

Additionally, we present simple odds ratios (OR) of emergence (odds to emerge in live against sterilized soil). OR > 1 indicate higher emergence in live than sterilized soil.

Biomass growth

We analysed seedling dry mass using generalized linear models (glm) and linear mixed effects models (lmer in lme4; Bates et al. 2015) separately for each species. We added the number of days between emergence and harvest of each seedling as a fixed effect to control for the effect of seedling age on biomass growth. Only seedlings ≥ 105 days since emergence were included (Appendix S1: Table S6). Seedling position in the shade house and seedling number per pot were considered as random effects and included in a model if they explained more than 5% of the variance.

For each model, we assessed the proportion of variance explained by the position of the seedling in the shade house and the number of seedlings growing in its pot by dividing the variance of the random effect by the total variance. A random effect was included in a model if it explained more than 5% of the variance. We added seedling position for *V. baccifera* (40% light, successional stage and association analyses), number of seedlings per pot for *S. pauciflora* (40% light, successional stage and association) and *A. membranacea* (40% light, successional stage and association), and both random effects for *A. membranacea* (5% light, association).

For *A. membranacea*, *G. superba*, and *F. occidentalis,* we present models including light as a factor, as well as separate models for seedlings growing under 40% and 5% light to facilitate comparisons among species. In the remaining species, all seedlings in the 5% light treatment died before the harvest precluding the analysis of the effect of light on biomass growth. We *a priori* defined inoculum, successional stage/association, and (where possible) light and the interaction between inoculum and successional stage/association as fixed effects. We log-transformed biomass growth data and tested for the normality of residuals, homoscedasticity and non-multicollinearity of predictors (DHARMa; Hartig 2022). We performed type III ANOVAs and calculated microbial effects as the difference in predicted mean biomass growth in live versus sterilized soils at each treatment level. We compared microbial effects among successional stage/association as described above.

Survival

We calculated the risk ratio (RR) of survival as the proportion of seedlings surviving in live versus sterilized soil for 415 days after sowing. A RR > 1 indicates a positive microbial effect and higher survival in live than sterilized soil. This basic analysis allowed for the inclusion of species-treatment combinations with 100% survival.

We also analysed the effect of time on the probability of survival in all species-treatment combinations where survival was not 100% using mixed effects Cox proportional hazards regression (coxme; Therneau 2024b). Cox models cannot compute survival probabilities if a species-treatment combination is without a single death event. As our goal was to compare survival between live and sterilized soil, we excluded species-treatment combinations without deaths and their corresponding live or sterilized treatment (*G. superba*: all treatments*; F.*

occidentalis: all stages under 40% light and 0-yr stage under 5% light). We included seedling position in the shade house as a random effect. We included inoculum, successional stage/association, and light, and the interactions between inoculum x successional stage/association and inoculum x light as fixed effects. The effects of inoculum and light varied over time, thereby violating the proportional hazard assumption (cox.zph in survival; Therneau 2024a). We thus split our data into time intervals of 100 days (timesplitter in Greg; Gordon and Seifert 2022) and included the interaction terms inoculum x time (all models except *F. occidentalis*) and light x time where necessary (all models except *F. occidentalis* and *S. pauciflora*). We fitted Kaplan-Meier survival curves (survfit in survminer; Kassambara, Kosinski, and Biecek 2021) including all main effects as described for the Cox models above.

We performed type III ANOVAs on our Cox models. For all species-treatment combinations for which Cox models could be performed, we assessed microbial effects as the hazard ratio (HR) of death in sterilized versus live soils for each successional stage/association under 40% and 5% light and the overall HR of growing under 40% versus 5% light using estimated marginal mean survival times (emmeans at response scale). A HR = 1 indicates equal survival in the compared treatments. A HR > 1 indicates a reduced mortality risk per unit time in live versus sterilized soil and thus a positive microbial effect. For light, HR > 1 represents better survival under 5% than 40% light. We calculated Bonferroni corrected pairwise contrasts of HR to identify differences in microbial effects among successional stage/association. Hazard ratios were translated into percentage change over time as %=(HR-1)*100.

RESULTS

Emergence

Emergence was lower in live than sterilized soils for four of the seven tree species and when averaged across species and light levels (Appendix S1: Table S6). This negative microbial effect on emergence was significant for two species (*A. membranacea*, *X. frutescens*), while the microbial effect was significantly positive in *V. baccifera* (Appendix S2: Table S1 & Fig. S1). We found microbial effects in at least one successional stage for five species and differences in microbial effects among successional stages in four species (Appendix S2: Fig. S1).

In four species, differences in microbial effects among successional stages were more pronounced under 5% light, while the opposite was the case for *A. membranacea* (Appendix S2: Fig. S1). These effects of light on the pairwise differences between microbial effects in different successional stages did not, however, translate into overall significant two- or three-way interactions between inoculum, successional stage, and light except for *V. baccifera* (Appendix S2: Table S1).

Averaged across species, microbial effects on emergence were positive in home soil but negative in away soil (Appendix S1: Table S6). Five species, with four significantly so, had more positive microbial effects in home than away soil (Appendix S1: Table S6). Two of them had a significant interaction of inoculum x association (*A. membranacea, X. frutescens*) and two others had a significant interaction of inoculum x association x light (*V. baccifera, F. occidentalis*; Appendix S2: Table S1) with a larger difference in microbial effects between home and away soil under 5% than under 40% light (Appendix S1: Table S6; Appendix S2: Fig. S1). In contrast, for *A.*

membranacea the difference between positive microbial effects in home and negative microbial effects in away soil was significant only at 40% light (Appendix S2: Fig. S1). *Xylopia frutescens* had less negative microbial effects in home than away soils and this effect was consistent across light levels (Appendix S1: Table S6; Appendix S2: Fig. S1).

Biomass growth

We found microbial effects on biomass growth in five of the seven species (Appendix S2: Table S2). All seven species had an overall higher biomass growth (on average 3.7 times higher) in live than sterilized soil under both light levels (Appendix S1: Table S6). This positive microbial effect was significant in at least one successional stage for five species: for four of seven species under 40% light and for two of three species under 5% light (Appendix S2: Fig. S2). We found no consistent interaction between microbiota and light, successional stage, or species' association respectively across species (Appendix S2: Table S2). Microbial effects differed with association only for *P. grandis,* which had more negative microbial effects in home than away soil (Appendix S2: Fig. S2). Light had a strong positive effect on the biomass growth of all three analysed species, that had ~3.5-, 10-, and 75-times higher biomass growth under 40% than 5% light respectively (Appendix S1: Table S6 & Appendix S2: Table S2).

Survival

Survival differed greatly among the species and was highest for the two late successional species *G. superba* (98.2%) and *F. occidentalis* (95.1%). Survival in the five species associated with the younger successional stages was on average 34.2% (range 2.9-63.1%; Appendix S1: Table S6).

We found significant microbial effects on survival in four of the six analysed species (Appendix S2: Table S3 & Fig. S3 - S4). While the direction of microbial effects varied among species (Appendix S2: Fig. S3), microbial effects averaged across species and treatments were positive with seedlings surviving 28% longer (mean HR =1.28; Appendix S2: Table S4) and having a 1.10-times higher chance of survival in live than in sterilized soil (mean RR =1.10; Appendix S1: Table S6). Microbial effects decreased with time for all four species for which this could be analysed (Appendix S2: Table S3).

We found no significant interaction between inoculum and successional stage effects on survival, and the interaction of inoculum and association was significant only for *A. membranacea* (Appendix S2: Table S3). However, four of the six analysed species showed significant differences between pairs of successional stages (Appendix S2: Fig. S3 & S4). The overall chance of survival was on average 1.07-times higher in live than sterilized soil for home soil and 1.13-times higher in live than sterilized soil for away soil (RR in Appendix S1: Table S6). Hazard ratios were generally more positive in home than away soil (Appendix S2: Table S3) and seedlings thus survived 83% and 11% longer in live than sterilized soil from home and away stages, respectively.

The difference in microbial effects on survival in home versus away soil varied with light level. Across species, the relative risk for survival was higher in home than away soil under 5% light but lower in home than away soil under 40% light. Microbial effects were significantly more

positive in home than away soil under 40% light in *P. grandis* and under 5% light in *V. baccifera* (Appendix S2: Fig. S3).

For four species, microbial effects varied significantly with light level (Appendix S2: Table S3 & Fig. S3). Across successional stages, microbial effects were more positive under 5% than 40% light for *S. pauciflora, A. membranacea*, and *X. frutescens*, but more negative under 5% than 40% light for *V. baccifera*. The effect of light on survival increased over time in four species and did not vary with time in two species (Appendix S2: Table S3).

Please see Appendix S2: Table S4 for an overview of all microbial effects and their contrasts.

Table S1. Summary statistics of seedling emergence of seven tree species. Seeds were germinated in live (including soil microbiota) or sterilized soils (“Inoculum”) collected from forests of four successional stages (Successional stage) under two light levels (Light). We show Chi-square and p-values for the analyses of variance (type III) on **(a) Successional stage** Bayesian generalized linear models. In separate analyses, we translated the four successional stages into the binary variable Association that compares the difference between successional stages at which a respective species is naturally abundant (home) versus successional stages in which the species is not abundant (away). We show the results of these **(b) Association** models (glm).

	Siparuna pauciflora		***Psychotria grandis***		***Vismia baccifera***		***Apeiba membranacea***		***Xylopia frutescens***		***Gustavia superba***		***Faramea occidentalis***		**All**
	χ^2	*p*	χ^2	*p*	χ^2	*p*	χ^2	*p*	χ^2	*p*	χ^2	*p*	χ^2	*p*	**df**
**(a) Successional stage**															
Inoculum	0.68	0.409	2.46	0.117	**14.56**	**<0.001**	**7.05**	**0.008**	**18.18**	**<0.001**	2.39	1.000	1.85	0.174	1
Successional stage	0.88	0.831	2.34	0.505	**10.18**	**0.017**	6.18	0.103	1.97	0.579	**9.72**	**0.021**	**11.98**	**0.007**	3
Light	0.81	0.367	0.36	0.548	**20.44**	**<0.001**	0.46	0.495	1.01	0.315	2.20	1.000	3.11	0.078	1
Inoculum x Successional stage	3.57	0.312	0.65	0.884	**11.66**	**0.009**	6.43	0.093	4.18	0.243	6.95	0.074	1.22	0.747	3
Inoculum x Light	0.07	0.786	2.17	0.141	**8.93**	**0.003**	**4.85**	**0.028**	1.50	0.221	2.44	1.000	1.12	0.291	1
Successional stage x Light	1.38	0.710	0.63	0.889	**15.33**	**0.002**	5.06	0.168	0.97	0.809	5.71	0.127	6.06	0.109	3
Inoculum x Successional stage x Light	2.17	0.539	2.29	0.514	**13.26**	**0.004**	4.41	0.221	0.90	0.825	5.54	0.136	7.74	0.052	3
**(b) Association**															
Inoculum	0.29	0.590	**7.39**	**0.007**	3.05	0.081	**7.31**	**0.007**	**30.43**	**<0.001**	0.32	0.572	**5.06**	**0.024**	1
Association	0.08	0.777	0.01	0.919	0.07	0.797	**3.95**	**0.047**	0.01	0.942	3.13	0.077	**8.65**	**0.003**	1
Light	0.04	0.832	0.08	0.772	0.03	0.864	0.21	0.644	0.03	0.859	0.53	0.468	0.10	0.754	1
Inoculum x Association	1.00	0.318	<0 .01	0.960	1.20	0.273	**4.60**	**0.032**	**3.80**	**0.050**	0.76	0.382	1.59	0.207	1
Inoculum x Light	0.02	0.894	0.19	0.659	1.40	0.236	**7.63**	**0.006**	0.01	0.937	< .01	0.978	0.12	0.724	1
Association x Light	0.47	0.491	0.59	0.441	**10.80**	**0.001**	3.48	0.062	0.05	0.828	0.50	0.480	<0 .01	0.990	1
Inoculum x Association x Light	0.05	0.831	2.38	0.123	**8.82**	**0.003**	3.67	0.055	0.06	0.808	< .01	0.977	**6.26**	**0.012**	1

Note: Degrees of freedom (df) were identical for all seven species and are presented in the rightmost column. Significant values ($p < 0.05$) indicated in bold.

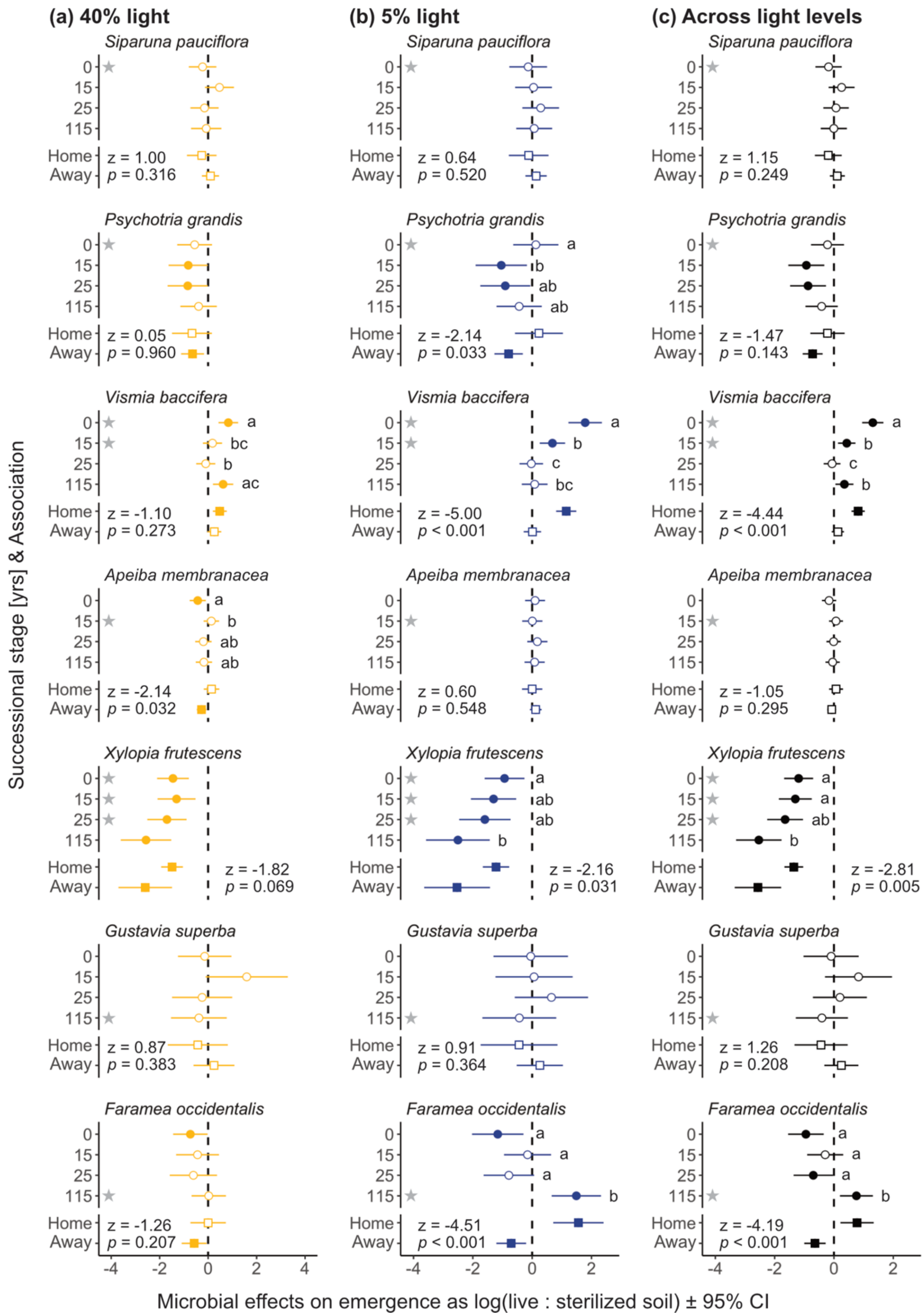

Figure S1. Caption printed on next page.

Figure S1. Microbial effects on seedling emergence in seven tree species. Seeds were germinated in live (including soil microbiota) or sterilized soils from forests of four successional stages (0, 15, 25 and 115 years of recovery). Additionally, we pooled all successional stages at which a species is abundant into home (grey stars) and all remaining stages into away. Microbial effects in four successional stages (circles) and in home versus away stages (squares) under **(a) 40% light**, **(b) 5% light**, and **(c) averaged across light** levels. Positive effects > 0 indicate more seedlings emerging in live than in sterilized soil. Filled symbols indicate significant effects ($p < 0.05$). Letters (successional stages) and z-ratios (home versus away) highlight significant differences between effects. For summaries of model results see Appendix S2: Tables S1-S3. For microbial effects and pairwise contrasts see Appendix S2: Table S4.

Table S2. Summary statistics of seedling biomass growth in seven tree species. Seedlings were grown under 40% or 5% light (Light) in live versus sterilized soils (Inoculum) collected from forests of four successional stages (Successional stage) that can be grouped into home and away stages (Association). We included seedling age to encompass the variation in the number of days between emergence and harvest per seedling. We show results of analyses of variance on linear mixed effects models for **(a) Successional stage** and **(b) Association** for seedlings of the seven species growing under 40% and 5% light.

	***Siparuna pauciflora* 40% light**		***Psychotria grandis* 40% light**		***Vismia baccifera* 40% light**		***Apeiba membranacea* 40% light**		***Apeiba membranacea* 5% light**	
**(a) Successional stage**	χ^2	**p**	χ^2	**p**	χ^2	**p**	χ^2	**p**	χ^2	**p**
Inoculum	4.32	**0.038**	4.56	**0.033**	25.52	**<0.001**	0.51	0.475	0.98	0.320
Successional stage	10.73	**0.013**	7.23	0.065	12.02	**0.007**	1.21	0.750	1.73	0.630
Inoculum x Succ. stage	1.41	0.493	8.46	**0.037**	0.17	0.676	1.52	0.677	7.92	**0.048**
Seedling age	0.65	0.419	12.79	**<0.001**	0.68	0.411	95.81	**<0.001**	137.49	**<0.001**
**(b) Association**										
Inoculum	12.73	**<0.001**	5.76	**0.016**	39.15	**<0.001**	0.33	0.566	1.21	0.271
Association	0.14	0.710	6.91	**0.009**	0.65	0.421	0.56	0.453	0.51	0.477
Inoculum x Association	<0.01	0.996	8.33	**0.004**	2.32	0.128	0.28	0.595	1.26	0.262
Seedling age	0.39	0.530	12.33	**<0.001**	0.09	0.759	46.32	**<0.001**	104.15	**<0.001**

Note: Significant values ($p < 0.05$) are indicated in bold.

Table S2 (continued)

	Xylopia frutescens 40% light		*Gustavia superba* 40% light		*Gustavia superba* 5% light		*Faramea occidentalis* 40% light		*Faramea occidentalis* 5% light	
**(a) Successional stage**	χ^2	**p**	χ^2	**p**	χ^2	**p**	χ^2	**p**	χ^2	**p**
Inoculum	7.27	**0.007**	2.12	0.146	0.16	0.689	9.37	**0.002**	0.80	0.370
Successional stage	2.02	0.569	6.24	**0.044**	0.99	0.803	2.65	0.449	2.41	0.493
Inoculum x Succ. stage	2.48	0.478	1.52	0.467	1.42	0.701	1.18	0.758	6.29	0.098
Seedling age	0.11	0.742	48.23	**<0.001**	38.55	**<0.001**	20.73	**<0.001**	4.35	**0.037**
**(b) Association**										
Inoculum	3.89	0.048	0.54	0.461	1.04	0.307	16.72	**<0.001**	6.03	**0.014**
Association	0.01	0.913	6.02	**0.014**	0.43	0.514	0.05	0.818	0.73	0.393
Inoculum x Association	0.60	0.439	0.33	0.569	1.33	0.249	0.04	0.839	2.80	0.094
Seedling age	0.16	0.688	60.11	**<0.001**	45.64	**<0.001**	12.85	**<0.001**	4.26	**0.039**

Table S2 (continued)

	Apeiba membranacea		*Gustavia superba*		*Faramea occidentalis*		**All**
**(a) Successional stage**	**χ^2**	**p**	**χ^2**	**p**	**χ^2**	**p**	**df**
Inoculum	0.01	0.933	2.36	0.125	15.61	**<0.001**	1
Successional stage	2.76	0.430	2.32	0.508	3.32	0.345	3
Light	14.85	**<0.001**	11.62	**0.001**	198.87	**<0.001**	1
Inoculum x Succ. stage	2.69	0.442	3.07	0.382	4.07	0.254	3
Inoculum x Light	0.10	0.756	0.92	0.337	5.35	**0.021**	1
Seedling age	113.87	**<0.001**	91.28	**<0.001**	21.28	**<0.001**	
**(b) Association**							
Inoculum	1.07	0.301	1.25	0.264	26.81	**<0.001**	1
Association	2.12	0.146	2.13	0.145	0.16	0.685	1
Light	24.29	**<0.001**	20.83	**<0.001**	199.26	**<0.001**	1
Inoculum x Association	2.53	0.111	1.20	0.273	0.58	0.445	1
Inoculum x Light	0.06	0.812	0.10	0.756	5.57	**0.018**	1
Seedling age	81.66	**<0.001**	92.88	**<0.001**	17.44	**<0.001**	

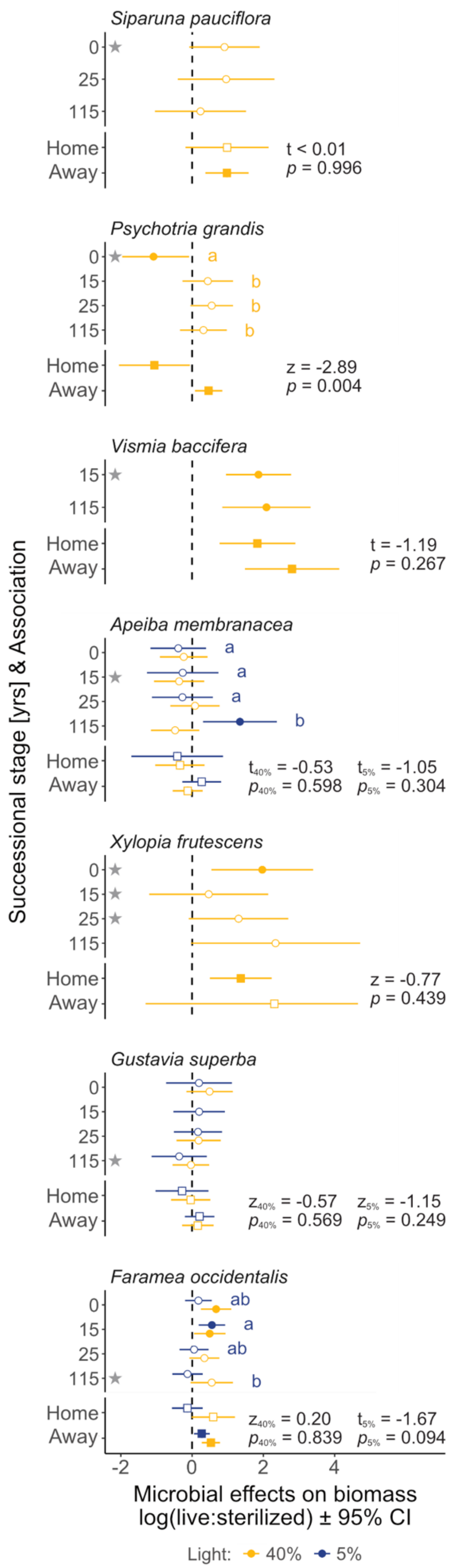

Figure S2. Microbial effects on seedling biomass growth. This is shown as difference in predicted mean biomass growth expressed in standard deviation units of biomass ± 95% confidence intervals in live versus sterilized soils from four successional stages (circles). Additionally, we show microbial effects in home (indicated by stars) and away soils (squares). Letters (successional stages) and z-ratios (home versus away) show significant contrasts between microbial effects at the $p < 0.05$ level. For a summary of the results of biomass growth models see Appendix S2: Table S2. For microbial effects and pairwise contrasts see Appendix S2: Table S4.

Table S3. Summary statistics for seedling survival in six tropical tree species. We assessed the probability of survival for the first 415 days after sowing in six tree species that were grown in live or sterilized soil ("Inoculum") collected from forests of four successional stages ("Successional stage") under two light levels ("Light"). We show results of type-III ANOVAs of mixed effects Cox proportional hazard models for separate analyses on **A) Successional stage** and **B) Association**. Association is a binary variable that pools successional stages into home stages, in which a species is naturally abundant and other stages (away).

	Siparuna pauciflora		***Psychotria grandis***		***Vismia baccifera***		***Apeiba membranacea***		***Xylopia frutescens***		***Faramea occidentalis***		**All**
	χ^2	***p***	χ^2	***p***	χ^2	***p***	χ^2	***p***	χ^2	***p***	χ^2	***p***	**df**
**A) Successional stage**													
Inoculum	**4.01**	**0.045**	**6.82**	**0.009**	**6.65**	**0.010**	**17.04**	**<0.001**	0.38	0.536	0.17	0.684	1
Successional stage	6.96	0.073	0.75	0.861	4.40	0.221	4.90	0.179	**8.21**	**0.042**	<0.01	1.000	3
Light	**19.52**	**<0.001**	<0.01	0.960	0.31	0.576	**9.69**	**0.002**	**74.68**	**<0.001**			1
Inoculum x Successional stage	1.91	0.592	3.77	0.287	2.89	0.408	5.25	0.155	1.02	0.796	0.01	0.997	3
Inoculum x Light	**21.02**	**<0.001**	0.19	0.664	**6.82**	**0.009**	**11.84**	**0.001**	**9.81**	**0.002**			1
Inoculum x Time	**-22.20**	**<0.001**	**-49.52**	**<0.001**	**- 155.41**	**<0.001**	**-3.94**	**0.047**					
Light x Time			**+55.15**	**<0.001**	**+69.43**	**<0.001**	**+147.92**	**<0.001**	**+7.35**	**0.007**			
**B) Association**													
Inoculum	**8.61**	**0.003**	**11.46**	**<0.001**	**25.92**	**<0.001**	**22.21**	**<0.001**	0.57	0.449	0.36	0.547	1
Association	2.49	0.114	0.10	0.757	1.09	0.296	3.21	0.073	**6.08**	**0.014**	0.10	0.747	1
Light	**19.52**	**<0.001**	<0.01	0.953	0.35	0.553	**9.61**	**0.002**	**72.40**	**<0.001**			1
Inoculum x Association	0.59	0.443	<0.01	0.955	2.35	0.125	**4.43**	**0.035**	<0.01	0.986	0.51	0.474	1
Inoculum x Light	**20.62**	**<0.001**	0.23	0.629	**5.55**	**0.018**	**11.97**	**0.001**	**9.21**	**<0.001**			1
Inoculum x Time	**-19.95**	**<0.001**	**-49.74**	**<0.001**	**- 166.66**	**<0.001**	-3.62	0.057	-0.39	0.530			
Light x Time			**+55.13**	**<0.001**	**+63.83**	**<0.001**	**+147.43**	**<0.001**	**+5.24**	**0.022**			

Note: For the interactive terms "inoculum x time" and "light x time" +/- symbols show derived model coefficients; "+" represents an increase of the effect of the variable with time and "-" a decrease in effect over time. Statistically significant values ($p < 0.05$) are indicated in bold.

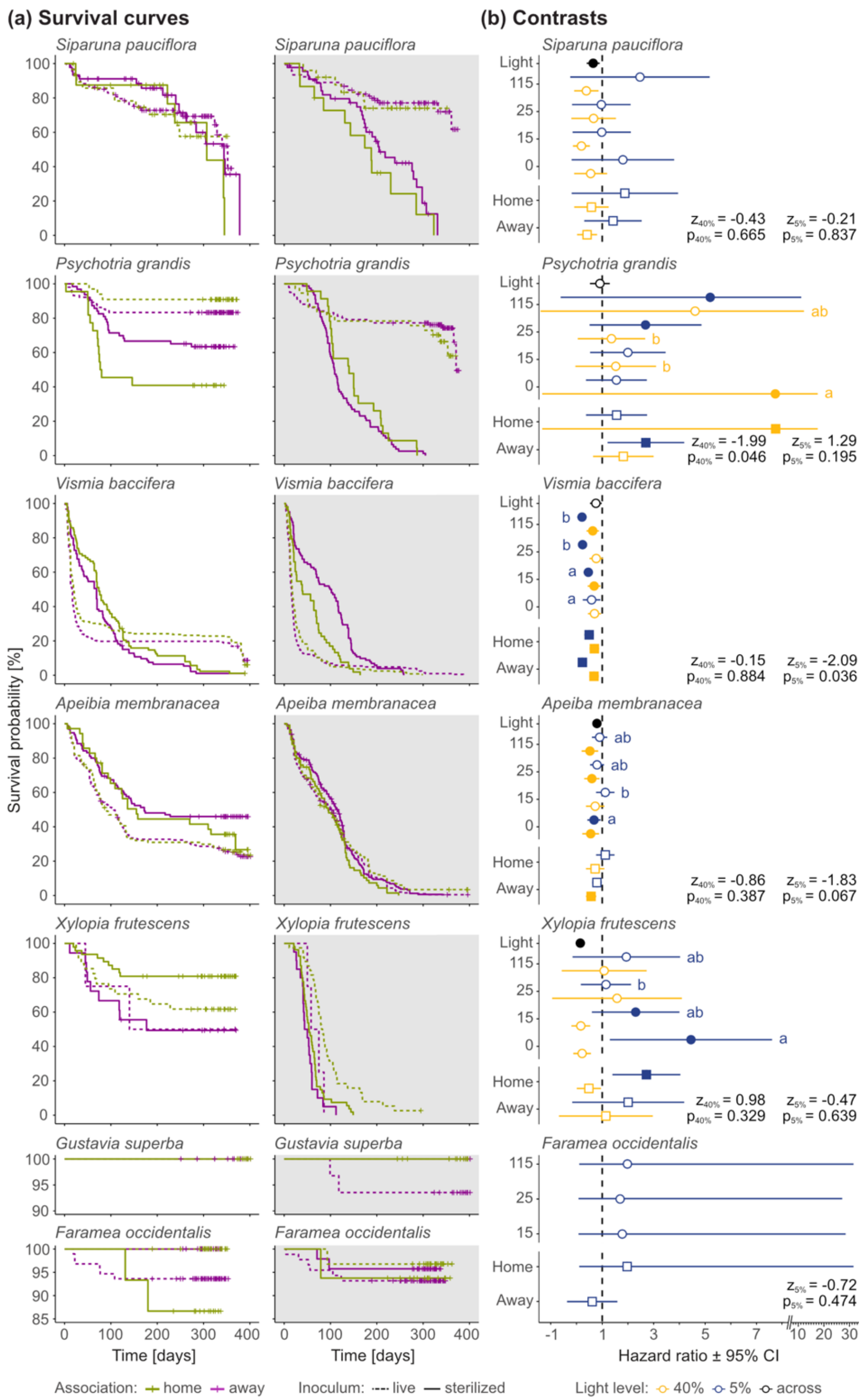

Figure S3. Caption printed on next page.

Figure S3. Seedling survival in seven tree species. (a) Survival probabilities over 415 days under 40% light (white background) and 5% light (grey background) in live (including microbiota) and sterilized soils. Forest soils of four successional stages were pooled into either home stages in which a species is abundant or other (away) stages. **(b) Contrasts** as hazard ratios (HR) are pairwise contrasts between the mortality risk when growing under 40% versus 5% light (Light) and in sterilized versus live soil for four successional stages (0, 15, 25 and 115 yrs) and for home and away stages. Filled symbols indicate HR that significantly differ from 1. A HR > 1 indicates positive microbial effects, i.e. higher survival in live versus sterilized soil or under 5% versus 40% light. Error bars represent 95% confidence intervals. Letters (successional stages) and z-ratios (home versus away) highlight significant differences between microbial effects within species-light combinations. For a summary of the results of survival models see Appendix S2: Table S3. For microbial effects and pairwise contrasts see Appendix S2: Table S4.

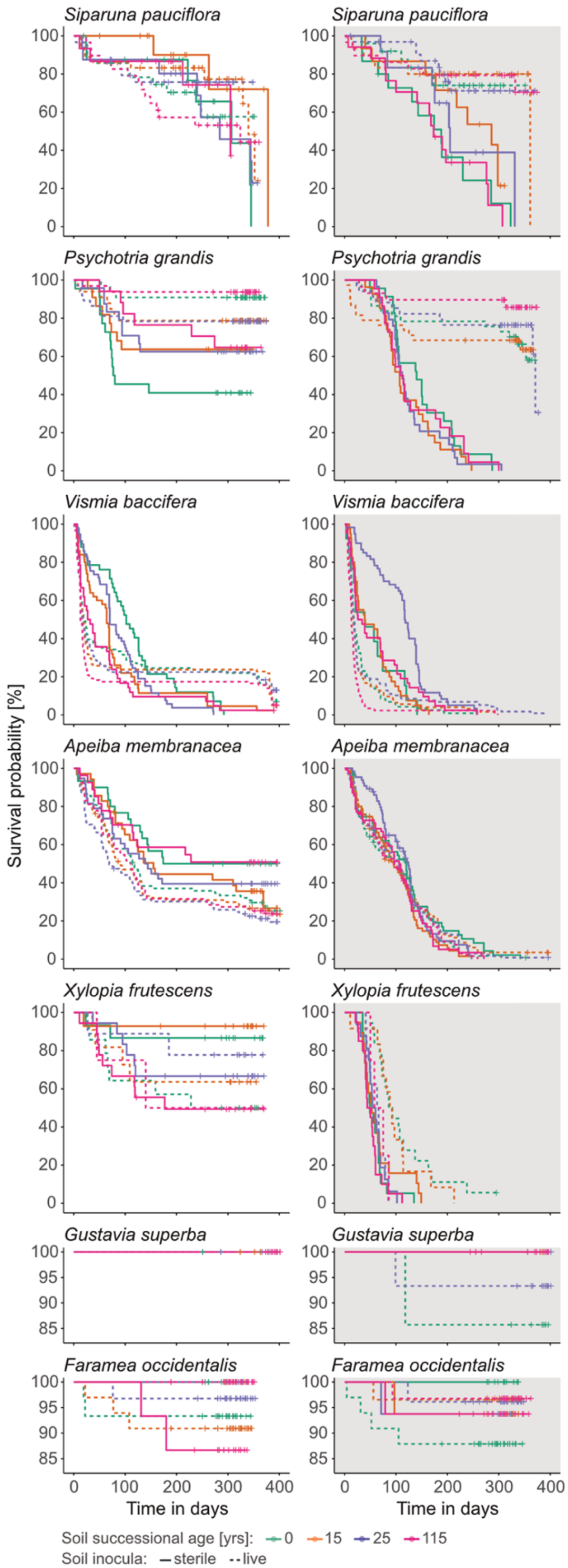

Figure S4. Kaplan-Meier curves of seedling survival probabilities of seven tree species in soils from four successional stages. Survival probabilities over 415 days under 40% light (white background) and 5% light (grey background) in live (including microbiota) and sterilized soils. Soils had been collected from forests of four successional stages (forests recovering for 0, 15, 25, and 115 years).

Table S4. Microbial effects on seedling performance and their pairwise contrasts. Microbial effects compare performance in sterilized versus live soils for **(a) Emergence** (logarithmic odds ratio, log(OR)) and **(b) Biomass growth** (estimate of difference in biomass growth) and **(c) Survival** (hazard ratio, HR), with 95% confidence intervals (95% CI). Seven tree species were grown under 40% and 5% daylight. We show pairwise contrasts between microbial effects at four successional stages and soils from home (pooling all stages at which a species is abundant) and other (away) stages.

	**(a) Emergence**				**(b) Biomass growth**				**(c) Mortality**			
	**40% light**		**5% light**		**40% light**		**5% light**		**40% light**		**5% light**	
	**log(OR)**	**95% CI**	**log(OR)**	**95% CI**	**est.**	**95% CI**	**est.**	**95% CI**	**HR**	**95% CI**	**HR**	**95% CI**
Siparuna pauciflora												
0	-0.23	-0.80 – 0.34	-0.13	-0.77 – 0.50	0.90	-0.09 – 1.89	–	–	0.55	0.17 – 1.77	1.80	0.60 – 5.45
15	0.47	-0.13 – 1.07	0.05	-0.57 – 0.67	–		–	–	0.19	0.03 – 1.07	0.98	0.31 – 3.11
25	-0.15	-0.73 – 0.43	0.29	-0.33 – 0.91	0.95	-0.41 – 2.30	–	–	0.66	0.18 – 2.45	0.97	0.30 – 3.14
115	-0.08	-0.71 – 0.55	0.07	-0.54 – 0.67	0.23	-1.05 – 1.51	–	–	0.38	0.11 – 1.32	2.47	0.82 – 7.41
Home	-0.26	-0.87 – 0.35	-0.12	-0.78 – 0.55	0.98	-0.19 – 2.15	–	–	0.58	0.18 – 1.84	1.88	0.63 – 5.64
Away	0.10	-0.26 – 0.45	0.13	-0.23 – 0.49	0.98	0.37 – 1.58	–	–	0.41	0.16 – 1.06	1.42	0.65 – 3.12
**Contrast**												
0-15	0.70	-0.11 – 1.51	0.18	-0.70 – 1.06	–	–	– –	–	2.87	0.46 – 17.68	1.84	0.45 – 7.49
0-25	0.08	-0.72 – 0.88	0.42	-0.45 – 1.30	0.05	-1.82 – 1.92	– –	–	0.83	0.20 – 3.45	1.87	0.45 – 7.84
0-115	0.15	-0.68 – 0.98	0.20	-0.66 – 1.07	-0.67	-1.98 – 0.63	– –	–	1.44	0.32 – 6.41	0.73	0.19 – 2.88
15-25	-0.62	-1.45 – 0.22	0.24	-0.63 – 1.12	–	–	– –	–	0.29	0.05 – 1.81	1.02	0.24 – 4.33
15-115	-0.55	-1.41 – 0.32	0.02	-0.84 – 0.89	–	–	– –	–	0.50	0.07 – 3.45	0.40	0.10 – 1.56
25-115	0.07	-0.78 – 0.92	-0.22	-1.08 – 0.64	-0.72	-2.99 – 1.55	– –	–	1.74	0.36 – 8.39	0.39	0.10 – 1.60
Away home	0.36	-0.35 – 1.07	0.25	-0.51 – 1.01	0.01	-1.28 – 1.29	– –	–	0.71	0.22 – 2.29	0.76	0.24 – 2.36

Table S4 (continued)

	(a) Emergence				(b) Biomass growth				(c) Mortality			
	40% light		5% light		40% light		5% light		40% light		5% light	
	log(OR)	95% CI	log(OR)	95% CI	est.	95% CI	est.	95% CI	HR	95% CI	HR	95% CI
Psychotria grandis												
0	-0.56	-1.28 – 0.16	0.12	-0.64 – 0.88	-1.09	-2.097 – -0.771	–	–	7.73	2.18 – 27.39	1.55	0.72 – 3.33
15	-0.82	-1.63 – -0.01	-1.04	-1.91 – -0.18	0.44	-0.284 – 1.153	–	–	1.53	0.55 – 4.26	2.00	0.96 – 4.16
25	-0.84	-1.67 – -0.01	-0.91	-1.75 – -0.06	0.54	-0.059 – 1.148	–	–	1.36	0.52 – 3.57	2.68	1.19 – 6.04
115	-0.39	-1.15 – 0.36	-0.44	-1.20 – 0.32	0.31	-0.352 – 0.976	–	–	4.61	0.92 – 23.04	5.20	1.68 – 16.12
Home	-0.67	-1.49 – 0.16	0.23	-0.58 – 1.04	-1.06	-2.05 – -0.06	–	–	7.74	2.19 – 27.44	1.56	0.73 – 3.34
Away	-0.64	-1.12 – -0.17	-0.79	-1.27 – -0.32	0.47	0.08 – 0.85	–	–	1.82	0.95 – 3.48	2.70	1.55 – 4.69
**Contrast**												
0-15	-0.26	-1.31 – 0.78	-1.16	-2.30 – -0.03	1.52	0.321 – 2.723	– –	–	5.06	1.01 – 25.49	0.78	0.30 – 2.02
0-25	-0.28	-1.34 – 0.77	-1.03	-2.14 – 0.09	1.63	0.475 – 2.788	– –	–	5.68	1.17 – 27.56	0.58	0.21 – 1.59
0-115	0.16	-0.84 – 1.17	-0.56	-1.62 – 0.50	1.40	0.201 – 2.597	– –	–	1.68	0.22 – 12.85	0.30	0.08 – 1.07
15-25	-0.02	-1.18 – 1.14	0.14	-1.07 – 1.34	0.11	-0.801 – 1.02	– –	–	1.12	0.28 – 4.52	0.74	0.27 – 2.02
15-115	0.43	-0.68 – 1.53	0.60	-0.55 – 1.75	-0.12	-1.087 – 0.842	– –	–	0.33	0.05 – 2.20	0.38	0.11 – 1.36
25-115	0.45	-0.67 – 1.57	0.47	-0.67 – 1.60	-0.23	-1.122 – 0.658	– –	–	0.30	0.05 – 1.90	0.52	0.14 – 1.88
Away home	0.02	-0.93 – 0.97	-1.02	-1.96 – -0.08	-1.52	-2.57 – -0.48	– –	–	0.24	0.06 – 0.96	1.73	0.76 – 3.92
Vismia baccifera												
0	0.83	0.43 – 1.24	1.79	1.22 – 2.35	–	–	–	–	0.70	0.49 – 1.01	0.59	0.33 – 1.05
15	0.18	-0.22 – 0.57	0.68	0.26 – 1.11	–	–	–	–	0.69	0.48 – 0.99	0.46	0.32 – 0.66
25	-0.10	-0.49 – 0.30	-0.03	-0.43 – 0.37	–	–	–	–	0.77	0.54 – 1.08	0.24	0.16 – 0.34
115	0.62	0.20 – 1.04	0.08	-0.35 – 0.52	–	–	–	–	0.63	0.43 – 0.93	0.21	0.14 – 0.32
Home	0.48	0.19 – 0.77	1.15	0.81 – 1.49	1.84	0.77 – 2.90	–	–	0.69	0.53 – 0.90	0.49	0.36 – 0.67
Away	0.26	-0.03 – 0.54	0.01	-0.28 – 0.30	2.81	1.48 – 4.13	–	–	0.68	0.53 – 0.88	0.23	0.17 – 0.31

Table S4 (continued)

	(a) Emergence				(b) Biomass growth				(c) Mortality			
	40% light		5% light		40% light		5% light		40% light		5% light	
	log(OR)	95% CI	log(OR)	95% CI	est.	95% CI	est.	95% CI	HR	95% CI	HR	95% CI
Vismia baccifera												
**Contrast**												
0-15	-0.65	-1.21 – -0.09	-1.10	-1.81 – -0.40	– –	–	– –	–	1.02	0.62 – 1.69	1.28	0.65 – 2.51
0-25	-0.93	-1.49 – -0.37	-1.82	-2.50 – -1.13	– –	–	– –	–	0.91	0.56 – 1.50	2.49	1.28 – 4.83
0-115	-0.21	-0.79 – 0.36	-1.70	-2.41 – -0.99	– –	–	– –	–	1.11	0.66 – 1.86	2.74	1.37 – 5.48
15-25	-0.28	-0.84 – 0.28	-0.71	-1.30 – -0.13	– –	–	– –	–	0.89	0.55 – 1.46	1.95	1.19 – 3.19
15-115	0.44	-0.14 – 1.01	-0.60	-1.21 – 0.01	– –	–	– –	–	1.09	0.66 – 1.79	2.15	1.27 – 3.63
25-115	0.72	0.14 – 1.29	0.11	-0.47 – 0.70	– –	–	– –	–	1.21	0.73 – 2.01	1.10	0.67 – 1.81
Away - home	-0.23	-0.64 – 0.18	-1.14	-1.59 – -0.69	-0.97	-2.85 – 0.91	–	–	0.98	0.69 – 1.39	0.46	0.31 – 0.69
Apeiba membranacea												
0	-0.43	-0.76 – -0.10	0.10	-0.24 – 0.43	-0.24	-0.91 – 0.43	-0.39	-1.23 – 0.44	0.55	0.31 – 1.00	0.68	0.47 – 1.00
15	0.13	-0.19 – 0.45	0.01	-0.33 – 0.35	-0.37	-1.07 – 0.33	-0.27	-1.34 – 0.80	0.73	0.44 – 1.19	1.12	0.81 – 1.54
25	-0.20	-0.53 – 0.14	0.17	-0.17 – 0.51	0.08	-0.61 – 0.77	-0.28	-1.19 – 0.64	0.60	0.36 – 0.98	0.80	0.57 – 1.12
115	-0.17	-0.51 – 0.16	0.08	-0.26 – 0.42	-0.48	-1.16 – 0.20	1.34	0.23 – 2.44	0.52	0.28 – 0.97	0.90	0.65 – 1.26
Home	0.14	-0.18 – 0.46	<.01	-0.34 – 0.34	-0.34	-1.03 – 0.35	-0.42	-1.70 – 0.87	0.73	0.44 – 1.19	1.12	0.82 – 1.54
Away	-0.27	-0.47 – -0.07	0.12	-0.08 – 0.32	-0.12	-0.54 – 0.30	0.27	-0.27 – 0.82	0.57	0.40 – 0.81	0.80	0.63 – 1.00
**Contrast**												
0-15	0.56	0.10 – 1.02	-0.09	-0.57 – 0.38	-0.13	-1.12 – 0.86	0.12	-1.21 – 1.46	0.76	0.36 – 1.58	-0.50	-0.95 – -0.04
0-25	0.23	-0.24 – 0.70	0.08	-0.40 – 0.55	0.32	-0.60 – 1.23	0.12	-1.09 – 1.33	0.92	0.44 – 1.92	-0.16	-0.62 – 0.31
0-115	0.26	-0.22 – 0.73	-0.02	-0.49 – 0.46	-0.24	-1.16 – 0.68	1.73	0.35 – 3.11	1.06	0.47 – 2.39	-0.28	-0.74 – 0.18
15-25	-0.33	-0.79 – 0.14	0.17	-0.31 – 0.65	0.45	-0.56 – 1.45	-0.01	-1.36 – 1.34	1.22	0.63 – 2.36	0.34	-0.09 – 0.76
15-115	-0.31	-0.77 – 0.16	0.08	-0.40 – 0.56	-0.11	-1.10 – 0.87	1.60	0.06 – 3.14	1.39	0.65 – 2.96	0.22	-0.21 – 0.65
25-115	0.02	-0.46 – 0.50	-0.09	-0.57 – 0.39	-0.56	-1.50 – 0.38	1.61	0.18 – 3.05	1.14	0.54 – 2.43	-0.12	-0.56 – 0.32
Away - home	-0.41	-0.79 – -0.04	0.12	-0.27 – 0.52	-0.22	-1.04 – 0.60	-0.69	-2.05 – 0.67	0.78	0.45 – 1.37	0.71	0.50 – 1.01

Table S4 (continued)

	(a) Emergence				(b) Biomass growth				(c) Mortality			
	40% light		5% light		40% light		5% light		40% light		5% light	
	log(OR)	95% CI	log(OR)	95% CI	est.	95% CI	est.	95% CI	HR	95% CI	HR	95% CI
Xylopia frutescens												
0	-1.45	-2.11 – -0.80	-0.93	-1.60 – -0.26	1.96	0.44 – 3.48	–	–	0.22	0.05 – 1.05	4.45	2.19 – 9.04
15	-1.30	-2.09 – -0.52	-1.30	-2.06 – -0.54	0.46	-1.32 – 2.24	–	–	0.17	0.02 – 1.50	2.30	1.10 – 4.82
25	-1.70	-2.51 – -0.89	-1.60	-2.46 – -0.73	1.30	-0.19 – 2.78	–	–	1.57	0.32 – 7.81	1.15	0.49 – 2.68
115	-2.56	-3.61 – -1.52	-2.50	-3.58 – -1.43	2.33	-0.19 – 4.86	–	–	1.08	0.23 – 4.98	1.94	0.66 – 5.69
Home	-1.49	-1.94 – -1.04	-1.22	-1.66 – -0.77	1.37	0.50 – 2.24	–	–	0.48	0.18 – 1.28	2.72	1.67 – 4.42
Away	-2.59	-3.70 – -1.49	-2.53	-3.64 – -1.42	2.31	-0.11 – 4.73	–	–	1.14	0.23 – 5.63	2.01	0.68 – 5.94
**Contrast**												
0-15	0.15	-0.85 – 1.14	-0.37	-1.37 – 0.63	-1.50	-3.80 – 0.80	–	–	1.31	0.09 – 19.40	1.94	0.71 – 5.24
0-25	-0.25	-1.26 – 0.77	-0.66	-1.75 – 0.42	-0.66	-2.78 – 1.46	–	–	0.14	0.01 – 1.31	3.88	1.28 – 11.75
0-115	-1.11	-2.31 – 0.08	-1.57	-2.82 – -0.32	0.37	-2.47 – 3.22	–	–	0.20	0.02 – 1.83	2.30	0.63 – 8.32
15-25	-0.40	-1.52 – 0.73	-0.29	-1.45 – 0.86	0.84	-1.48 – 3.15	–	–	0.11	0.01 – 1.60	2.01	0.65 – 6.19
15-115	-1.26	-2.56 – 0.04	-1.20	-2.52 – 0.11	1.87	-1.14 – 4.88	–	–	0.16	0.01 – 2.26	1.19	0.32 – 4.38
25-115	-0.86	-2.18 – 0.45	-0.91	-2.29 – 0.47	1.04	-1.88 – 3.96	–	–	1.47	0.16 – 13.43	0.59	0.15 – 2.33
Away - home	-1.10	-2.30 – 0.09	-1.31	-2.51 – -0.12	-0.94	-3.45 – 1.57	–	–	2.38	0.41 – 13.70	0.73	0.23 – 2.41
Gustavia superba												
0	-0.14	-1.24 – 0.96	-0.05	-1.31 – 1.21	0.48	-0.19 – 1.16	0.19	-0.76 – 1.14	–	–	–	–
15	1.59	-0.11 – 3.29	0.06	-1.24 – 1.36	–	–	0.19	-0.55 – 0.94	–	–	–	–
25	-0.25	-1.49 – 0.99	0.65	-0.59 – 1.88	0.18	-0.46 – 0.82	0.16	-0.53 – 0.85	–	–	–	–
115	-0.38	-1.53 – 0.77	-0.43	-1.68 – 0.81	-0.04	-0.57 – 0.49	-0.37	-1.16 – 0.43	–	–	–	–
Home	-0.43	-1.66 – 0.81	-0.44	-1.74 – 0.85	-0.04	-0.56 – 0.49	-0.28	-1.03 – 0.46	–	–	–	–
Away	0.24	-0.60 – 1.09	0.26	-0.52 – 1.04	0.32	-0.13 – 0.77	0.21	-0.20 – 0.63	–	–	–	–

Table S4 (continued)

	(a) Emergence				(b) Biomass growth				(c) Mortality			
	40% light		5% light		40% light		5% light		40% light		5% light	
	log(OR)	95% CI	log(OR)	95% CI	est.	95% CI	est.	95% CI	HR	95% CI	HR	95% CI
Gustavia superba												
**Contrast**												
0-15	1.73	-0.11 – 3.58	0.11	-1.63 – 1.86	–	–	0.01	-1.20 – 1.21	–	–	–	–
0-25	-0.11	-1.65 – 1.44	0.70	-1.00 – 2.40	-0.31	-1.25 – 0.64	-0.03	-1.20 – 1.14	–	–	–	–
0-115	-0.24	-1.73 – 1.25	-0.38	-2.09 – 1.32	-0.52	-1.38 – 0.34	-0.56	-1.82 – 0.71	–	–	–	–
15-25	-1.84	-3.90 – 0.23	0.59	-1.20 – 2.37	–	–	-0.03	-1.05 – 0.98	–	–	–	–
15-115	-1.97	-3.99 – 0.04	-0.50	-2.29 – 1.30	–	–	-0.56	-1.65 – 0.53	–	–	–	–
25-115	-0.13	-1.81 – 1.54	-1.08	-2.83 – 0.66	-0.22	-1.05 – 0.62	-0.53	-1.59 – 0.53	–	–	–	–
Away - home	0.67	-0.83 – 2.17	0.70	-0.81 – 2.21	-0.36	-1.05 – 0.33	-0.49	-1.35 – 0.37	–	–	–	–
Faramea occidentalis												
0	-0.73	-1.44 – -0.02	-1.16	-2.03 – -0.29	0.67	0.24 – 1.10	0.17	-0.21 – 0.55	–	–	–	
15	-0.44	-1.32 – 0.45	-0.16	-0.95 – 0.64	0.49	0.04 – 0.93	0.55	0.18 – 0.93	–	–	1.78	0.11 – 28.40
25	-0.61	-1.58 – 0.37	-0.79	-1.64 – 0.06	0.34	-0.08 – 0.76	0.05	-0.36 – 0.46	–	–	1.70	0.11 – 27.10
115	0.03	-0.68 – 0.73	1.49	0.66 – 2.32	0.54	-0.05 – 1.14	-0.13	-0.57 – 0.30	–	–	1.98	0.12 – 31.60
Home	<.01	-0.73 – 0.73	1.56	0.71 – 2.41	0.60	-0.01 – 1.21	-0.13	-0.56 – 0.30	–	–	1.97	0.12 – 31.47
Away	-0.58	-1.09 – -0.06	-0.71	-1.21 – -0.21	0.53	0.27 – 0.78	0.27	0.05 – 0.50	–	–	0.61	0.12 – 3.03
**Contrast**												
0-15	0.30	-0.80 – 1.39	1.01	-0.15 – 2.16	-0.18	-0.80 – 0.44	0.38	-0.15 – 0.91	–	–	–	–
0-25	0.12	-1.04 – 1.28	0.37	-0.81 – 1.56	-0.33	-0.93 – 0.28	-0.12	-0.68 – 0.44	–	–	–	–
0-115	0.76	-0.22 – 1.73	2.65	1.47 – 3.83	-0.12	-0.86 – 0.61	-0.31	-0.88 – 0.27	–	–	–	–
15-25	-0.17	-1.48 – 1.14	-0.63	-1.79 – 0.53	-0.15	-0.76 – 0.47	-0.50	-1.06 – 0.06	–	–	1.05	0.02 – 52.90
15-115	0.46	-0.67 – 1.59	1.65	0.50 – 2.79	0.06	-0.69 – 0.80	-0.69	-1.26 – -0.11	–	–	0.90	0.02 – 45.30
25-115	0.63	-0.57 – 1.84	2.28	1.09 – 3.46	0.21	-0.53 – 0.94	-0.19	-0.77 – 0.40	–	–	0.86	0.02 – 43.20
Away - home	-0.58	-1.47 – 0.32	-2.27	-3.25 – -1.38	0.07	-0.59 – 0.73	-0.41	-0.89 – 0.08	–	–	0.31	0.01 – 7.62

References

Bates, Douglas, Martin Maechler, Ben Bolker, and Steve Walker. 2015. "Fitting Linear Mixed-Effects Models Using Lme4." *Journal of Statistical Software* 67 (1): 1–48. https://doi.org/10.18637/jss.v067.i01.

Burnham, Kenneth P., and David R. Anderson. 2004. "Multimodel Inference: Understanding AIC and BIC in Model Selection." *Sociological Methods & Research* 33 (2): 261–304. https://doi.org/10.1177/0049124104268644.

Fox, John, and Sanford Weisberg. 2019. *An R Companion to Applied Regression*. 3rd ed. Sage. https://www.john-fox.ca/Companion/.

Gelman, A., and Y. Su. 2022. *Arm: Data Analysis Using Regression and Multilevel/Hierarchical Models*. V. 1.13-1. Released. https://CRAN.R-project.org/package=arm.

Gordon, M., and R. Seifert. 2022. *Greg: Regression Helper Functions*. V. 1.4.1. Released. https://CRAN.R-project.org/package=Greg.

Hartig, F. 2022. *DHARMa: Residual Diagnostics for Hierarchical (Multi-Level / Mixed) Regression Models*. Released. https://CRAN.R-project.org/package=DHARMa.

Kassambara, Alboukadel, Marcin Kosinski, and Przemyslaw Biecek. 2021. *Survminer: Drawing Survival Curves Using "Ggplot2".* V. version 0.4.9. Released. https://CRAN.R-project.org/package=survminer.

Lenth, R. 2022. *Emmeans: Estimated Marginal Means, Aka Least-Squares Means*. V. 1.8.1-1. Released. https://CRAN.R-project.org/package=emmeans.

Lüdecke, Daniel, Mattan S. Ben-Shachar, Indrajeet Patil, Philip Waggoner, and Dominique Makowski. 2021. "Performance: An R Package for Assessment, Comparison and Testing of Statistical Models." *Journal of Open Source Software* 6 (60): 3139. https://doi.org/10.21105/joss.03139.

R Core Team. 2024. *R: A Language and Environment for Statistical Computing*. R Foundation for Statistical Computing, released. https://www.R-project.org/.

Therneau, TM. 2024a. *A Package for Survival Analysis in R*. V. 3.7-0. Released. https://CRAN.R-project.org/package=survival.

Therneau, TM. 2024b. *Coxme: Mixed Effects Cox Models*. V. 2.2-22. Released. https://CRAN.R-project.org/package=coxme.
